# Investigating the Evidence of Behavioral, Cognitive, and Psychiatric Endophenotypes in Autism: A Systematic Review

**DOI:** 10.1155/2017/6346912

**Published:** 2017-07-05

**Authors:** Kavita Ruparelia, Karim Manji, Amina Abubakar, Charles R. Newton

**Affiliations:** ^1^Open University, Milton Keynes, UK; ^2^Department of Pediatrics and Child Health, Muhimbili University of Health and Allied Sciences, Dar es Salaam, Tanzania; ^3^KEMRI-Wellcome Trust Research Programme, Centre for Geographic Medicine Research (Coast), Kilifi, Kenya; ^4^Department of Psychiatry, University of Oxford, Oxford, UK

## Abstract

Substantial evidence indicates that parents of autistic individuals often display milder forms of autistic traits referred to as the broader autism phenotype (BAP). To determine if discrete endophenotypes of autism can be identified, we reviewed the literature to assess the evidence of behavioral, cognitive, and psychiatric profiles of the BAP. A systematic review was conducted using EMBASE, MEDLINE, PsycINFO, PsycEXTRA, and Global Health. Sixty papers met our inclusion criteria and results are discussed according to the proportion of studies that yield significant deficits per domain. The behavioral, cognitive, and psychiatric endophenotypes in parents of autistic probands are still not clarified; however, evidence suggests mild social/communication deficits, rigid/aloof personality traits, and pragmatic language difficulties as the most useful sociobehavioral candidate endophenotype traits. The existence of deficits in the cognitive domain does suggest familial vulnerability for autism. Furthermore, increased depressed mood and anxiety can also be useful markers; however, findings should be interpreted with caution because of the small number of studies in such heterogeneously broad domains and several methodological limitations.

## 1. Introduction

Autism is a life-long complex neurodevelopmental disorder which has heterogeneous clinical manifestations and multifactorial aetiology. It is characterized by impairments in social interaction and communication and restricted patterns of behavior, interests, and activities, occurring within the first 3 years of life [1].

The heritability of autism is estimated to be from 70% to 90% [2, 3]. Research suggests the risk of developing autism in siblings of individuals with autism is between 10 and 20%, considerably higher than when compared to about 1% for siblings of typically developing children [4, 5]. These data suggest a strong genetic basis, despite the clinical heterogeneity. Since numerous studies using linkage or candidate gene approaches have not discovered a single genetic locus of major effect, it is thought that the definition of the endophenotypes may provide insights into the biological basis of this condition.

Studies have provided substantial evidence indicating that first-degree relatives of autistic individuals often display milder forms of autistic traits referred to as the broader autism phenotype (BAP) [6]. This milder expression includes a set of behavioral and cognitive characteristics that reflect the phenotypic expression that is qualitatively similar in unaffected relatives of autistic individuals. For instance, mild challenges in social cognition in using facial cues and other features to determine mental states have been noted in parents of children with autism [7]. Additional studies report similar differences in emotion processing abilities, particularly emotion identification [8, 9] and phonological processing [10]. Research that includes such quantitative measures of autistic traits and underlying mechanisms responsible for such features in first-degree relatives is fundamental in studying the genetic basis of autism as it can help to identify which characteristics aggregate in family members and are thus likely to be potential endophenotypes for autism at the neurocognitive level.

Endophenotypes are heritable markers associated with a given condition and can provide insight into its etiology. Gottesman and Gould [11] offered a set of criteria for identification of useful endophenotypes suggesting that deficits must be (a) associated with illness in the population; (b) heritable; (c) state-independent (manifesting in an individual whether or not illness is active); (d) cosegregated with the condition within families; and (e) also found in unaffected relatives at a higher prevalence than in the general population. The study of endophenotypes is particularly useful in understanding developmental disorders such as autism that are diagnosed on clinical features but are of neurobiological origin and can aid to better identify and characterize the nature of the genetic contributions to this complex disorder.

Several researchers have reviewed the BAP traits in first-degree relatives of autistic probands [12–14]. Some reviews include studies that have examined the BAP in parents and siblings of autistic probands. Although features of the autism phenotype have been found in the “at risk” infant sibling studies, no clear distinction can be made to determine whether they are the characteristics of the BAP or whether the infant siblings may later receive an autism diagnosis. Thus, we limited this review process to parents only by employing a systematic approach to focus on the sociobehavioral, cognitive and psychiatric profiles of the broader autism phenotype to determine candidate endophenotypic traits for autism.

We conducted a systematic review of the literature to assess the evidence of behavioral, cognitive, and psychiatric endophenotypes of autism in parents. The aim of this review was to ascertain whether parents of probands with autism have higher prevalence of various components of the BAP and more specifically of behavioral, cognitive, and other psychiatric conditions. The questions addressed were as follows:

1. What are the behavioral, cognitive, and other psychiatric (focusing primarily on depression and anxiety) endophenotypes of autism as manifested through the broader autism phenotype in biological parents of autistic probands?
2. What are the tools used to measure these endophenotypes and the magnitude of effect?
3. Do patterns evident in endophenotypes of autism provide insight into cultural and geographical differences?

## 2. Review Methods

### 2.1. Data Sources and Search Strategy

**Figure 1:**
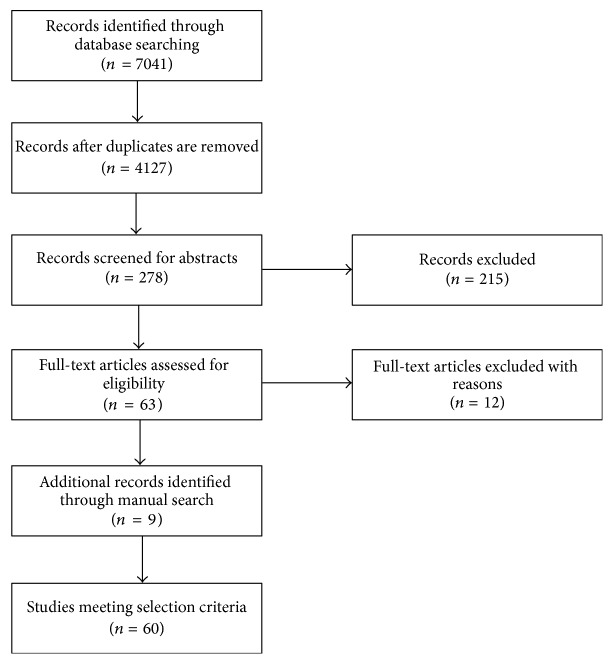
Flow chart of study selection.

A comprehensive literature search was performed to collate evidence of behavioral, cognitive, and psychiatric endophenotypes in autism. Literature searches for published and grey literature were subsequently carried out using 5 databases, EMBASE, MEDLINE, PsycINFO, PsycEXTRA, and Global Health, from inception to August 2014 without language restriction. The strategy was developed by breaking down the review questions into elemental facets according to the recommendations of the National Health Service Centre for Reviews and Disseminations [15]. These facets included exposure, outcome, population, publication language, and keywords (Table 1). The initial search strategy used the words “autis^*∗*^ AND endophenotyp^*∗*^ OR phenotyp^*∗*^”. These searches were further refined by the addition of the outcome terms and population (“parent^*∗*^ OR relative OR famil^*∗*^”). The bibliographies of key references were later hand-searched to identify articles missed in the database search.  Figure 1 illustrates our literature search strategy.

### 2.2. Data Selection Criteria

The titles and abstracts of papers identified were reviewed and the full versions of potential papers were read to decide on final selection. The inclusion criteria were

1. original studies that employed a quantitative methodological approach to investigate behavioral, cognitive, and psychiatric (depression and anxiety) endophenotypes in biological parents,
2. the fact that autistic proband (other conditions on the spectrum such as Asperger Syndrome, Pervasive Developmental Disorder, and Pervasive Developmental Disorder Not Otherwise Specified were also included) must have a clinically established diagnosis of autism (minimum DSM III) and no concomitant medical conditions associated with autistic symptomatology and visual, auditory, and motor impairment such as Fragile X or Tuberous Sclerosis.
3. Studies that carried out a comparison of endophenotypes between parents of individuals diagnosed with autism and unaffected adults, a normative parental control group, and/or a clinical parental control group.

We excluded any studies investigating the BAP in the general population, studies on genetics and autism, and studies examining the neuroanatomical and neurofunctional dimensions of the BAP. All single case studies, case series, book chapters, theoretical papers, review papers, unpublished dissertations/theses, and studies not published in English were excluded.

The final set of papers was restricted to those that quantitatively evaluated behavioral, cognitive, and psychiatric endophenotypes in biological parents of autistic probands.

### 2.3. Data Extraction

The author (KR) examined the titles, abstracts, and studies with study selection criteria. Data were organized into broad domains for each of the three categories: sociobehavioral, that is, direct assessment of BAP expression, other measures of personality and friendships, social interaction, repetitive/restrictive interests, and social and narrative language; cognitive, that is, intellectual functioning, structural language, social cognition, executive function, local visual processing (central coherence), and visual perception; other psychiatric conditions, specifically depression and anxiety.

### 2.4. Effect Sizes

The data extracted was based on heterogeneous measures and outcomes, so pooling the data in a meta-analysis was inappropriate. To compare the robustness of the measures used, for each behavioral, cognitive, and psychiatric variable of interest an effect size (ES) was computed from the data reported in each study. Cohen's effect size statistic (*d*) was calculated as the difference between the means of both groups divided by the pooled standard deviation. The following criteria were used to assess the magnitude of effect: *d* < 0.2 (small), *d* > 0.5 (medium), and *d* > 0.8 (large) [16].

## 3. Results

### 3.1. Search Results

The initial electronic search identified 7,041 records, of which 4,127 records remained after duplicates were removed. 278 articles were eligible for full review after examination of titles and abstracts (Figure 1). After full text review, we excluded 12 articles for the following reasons: in 9 studies it was not possible to distinguish parent and sibling data when results were reported for combined first-degree relatives, and, in 3 studies, proband diagnosis was established using criteria prior to DSM III. The search criteria, additional articles identified through manual search, and total numbers of articles meeting selection criteria are shown in Figure 1.

### 3.2. Results of Literature Extraction

Twenty-five of the 60 studies that fulfilled the inclusion criteria directly evaluated the BAP expression (including personality, social behavior, and pragmatic language features of the BAP). An additional 7 studies assessed other aspects within the sociobehavioral domain. Thirty-seven reports assessed the broad domain of cognitive functioning and seven studies investigated other psychiatric conditions. Twenty-seven of the studies were conducted in North America, 24 in Western Europe, 4 in the Middle East, and 3 in Western Pacific and 1 was conducted in South America and 1 used combined samples from North America, Western Europe, and Western Pacific. However, no studies were conducted in Asia or Africa. Index families included a total of 4,833 mothers and 3,065 fathers that took part across all studies reviewed (few studies did not specify sex breakdown). Studies varied greatly in their choice of comparison control group, with 26 studies using a nonclinical comparison group, 21 studies using a normative control sample, and 13 studies using a combined sample of clinical and nonclinical control groups. Thirteen studies evaluated the gradation of expression across family types using families with multiple incidence autism (MPX) and single incidence autism (SPX).

We summarized the results of the literature search according to different sociobehavioral, cognitive and psychiatric domains. For each domain we present the measures used within that domain and any significant differences found between index parents and parental controls, and so results are described in relation to proband diagnosis. All background measures used to establish BAP status without using a comparison group as well as control tasks are not reported under the specific criteria in this review.

### 3.3. Sociobehavioral Domain (Supplementary Table 1)

This domain includes studies that evaluated the BAP expression using measures designed specifically to assess social abilities, communication skills, and personality traits characteristic of the BAP, as well as measures of reciprocal interaction, restrictive, and repetitive interest and social and narrative language.

#### 3.3.1. BAP Expression through Direct Clinical Assessment

Studies explored the BAP using a variety of measures and research designs with some studies utilizing conservative selection criteria, dividing parents of autistic probands into “BAP present” (BAP+) and “BAP absent” (BAP−) groups. As shown in Supplementary Table 1 in Supplementary Material available online at https://doi.org/10.1155/2017/6346912, from eight of the measures specifically designed to assess the BAP, four are more recent questionnaires aiming to assess the BAP quantitatively, and four use interviews and direct behavioral observations. Of the four questionnaires, one is a self-report measure (Autism Spectrum Quotient (AQ)), two are informant report measures (Communication Checklist-Adult (CCA); and Social Responsiveness Scale (SRS)), and one is a self-report and informant report questionnaire (Broader Autism Phenotype Questionnaire (BAPQ)). Of the four remaining measures, two are semistructured interviews (Family History Interview (FHI)/Family History Schedule (FHS) and Modified Personality Assessment Schedule (MPAS)/Modified Personality Assessment Schedule-Revised (MPAS-R)) and two assess BAP via interviews and direct clinical observation/assessment (Broader Phenotype Autism Symptom Scale (BPASS) and Pragmatic Rating Scale (PRS)).

*Autism Spectrum Quotient (AQ)*. A total of ten reports measured the BAP using the self-report AQ (ES range: 0.01–1.34). Three studies used adaptations of the AQ: one in Italian [17], one in Turkish [18], and one in French [19]. Within the “social skills” factor, five studies found significantly higher deficits in social skills compared to parents of typically developing children [17, 18, 20–22]. Two studies reported significantly higher prevalence of “Attention Switching” deficits between the index parents and parents of typically developing children [22] and parents of children with specific language impairment [23]. One study evaluating the “Attention to Detail” subscale reported mothers of typically developing children scoring significantly higher than index mothers [24]. Within the “Communication” subscale, five out of eight studies reported significantly higher communication deficits between index parents and parents of typically developing children [17, 18, 20, 22] and parents of children with a specific language impairment [23]. However, only Wheelwright et al.'s (2010) [22] study reported a significant trend for index parents to have more deficits in “Imagination” subscale compared to a sample of parents of typically developing children. For the total AQ score, four studies reported higher combined total scores among index parents when compared to parents of typically developing children [17, 18, 22] and parents of children with specific language impairment [23].

Ingersoll et al. (2011) [25] combined the social skill and communication factors and revealed index mothers to score significantly higher than normative mothers on the AQ. Furthermore, in a more recent study, using a validated French Autism Quotient (FAQ), Robel et al. (2014) [19] distributed AQ scores between two main factors, F1 corresponding to socialization and communication and F2 corresponding to imagination and rigidity. They reported index parents to have more symptomatic scores in the F1 domain compared to parents of typically developing children. No significant differences were found for the F2 domain; however, the global score (F1 and F2 combined) remained significant with index parents scoring higher.

*Broader Autism Phenotype Questionnaire (BAPQ)*. Two studies evaluated the BAP using the BAPQ (ES range: 0.26–1.49). Hurley et al. (2007) [26] used the method of preestablishing parents of autistic probands into “BAP present” (BAP+) and “BAP absent” (BAP−) groups by direct assessment on MPAS-R and PRS, reporting consistently higher scores for “BAP+” group compared to “BAP−” group and community control parents on all subscales: aloof, rigid, pragmatic language, and the total score. More recently, Sasson et al. (2013) [27] reported similar results for all BAPQ subscales and total score, with index fathers scoring significantly higher than normative fathers, and the same trend was significant for mothers of both groups. 

*Broader Phenotype Autism Symptom Scale (BPASS)*. Bernier et al. (2012) [28] used the BPASS to assess the BAP in MPX parents compared to parents of SPX families, parents of developmentally delayed children, and parents of typically developing children (ES range 0.75–1.28). Differences among groups were found in the “Social Motivation” subscale where MPX parents showed significantly more deficits than the SPX parents, parents of developmentally delayed children, and parents of typically developing children. In both “Expressiveness” and “Restricted Interests” subscales a significant difference was found only between the MPX parents scoring higher than parents of typically developing children. No group differences were found within the “Communication” subscale and, interestingly, SPX parents did not differ from parents of children with developmental delay or typical development. 

*Communication Checklist-Adult Version (CC-A)*. Whitehouse et al. (2010) [29] assessed the BAP using the CC-A (ES range: 0.04–0.43), and found only the “Social Engagement” subscale had statistically significant differences between the index parents and a normative sample, suggesting a more passive communication style for the index parents. No group differences were found in the “Language Structure” and “Pragmatic Language” subscales; however, analysis of the total score of the two groups (1 standard deviation below mean) was found to be significant.

*Family History Interview/Family History Schedule (FHI/FHS)*. Three studies evaluated the BAP using the FHI/FHS semistructured interview method (no ES available). Folstein et al. (1999) [30] analyzed four items (language delays, reading difficulties, spelling difficulties, and articulation) on the “Communication” subscale. Accordingly, “early language-related cognitive difficulties” (ELRCD) were scored and a “definite” or “probable” rating was applied. Significantly higher rates of definite and probable ELRCD were found in index parents compared to parents of children with Down's Syndrome. However, two other studies found index parents to perform equally to comparison groups on the “Communication” subscale [6, 31]. Within the “social” factor, Piven et al. (1997) [6] found parents from MPX families had significantly higher prevalence of social deficits than parents of Down's Syndrome children, particularly in index fathers. Similarly, Pickles et al. (2013) [31] reported significantly increased social deficits in index parents compared to parents of children with a specific language impairment. Interestingly, no group differences were found between index parents and parents of children with a combined diagnosis of specific language impairment and autism. Only Piven et al. (1997) [6] assessed the “Stereotyped Behaviors” subscale and reported MPX parents to have significantly more repetitive stereotyped behaviors compared to parents of Down's Syndrome children.

*Modified Personality Assessment Schedule (MPAS/MPAS-R)*. One study used the MPAS to evaluate the BAP (Piven et al., 1994) [32] and three subsequent studies have used a modified version (MPAS-R) [33–35] (ES not available). Three out of the four studies assessing the “Aloof” subscale found significantly higher rates of aloofness in index parents compared to parents of Down's Syndrome children [32, 33], with one study reporting MPX parents to score significantly higher than SPX parents who in turn scored significantly higher than parents of children with Down's Syndrome [35]. Similarly, the same trend for the “Anxious,” “Hypersensitive,” “Rigid,” and “Untactful” personality traits was reported [35]. Piven et al. (1997) [33] reported significantly higher rates of anxiousness, hypersensitiveness, and rigidity in MPX parents in comparison to parents of Down's Syndrome; however, they found no significant differences between the two groups in the “Untactful,” “Undemonstrative,” and “Unresponsive” traits. Piven et al. (1994) [32], however, did find significantly higher rates of untactfulness and undemonstrativeness in index parents compared to parents of children with Down's Syndrome. In a more recent study, Losh et al. (2012) [35] failed to find a significant difference for the “Overly Conscientious” subscale, but they did find a significant difference in the “Rigidity” subscale. 

*Pragmatic Rating Scale (PRS/PRS-M)*. A total of five studies assessed the BAP using the PRS (ES range: 0–1.14). Landa et al. (1992) [36] combined blind and nonblind ratings and reported higher total scores for the index parents compared to their control sample of parents of Down's Syndrome and typical development. Losh et al. (2012) [35] found in their sample of mothers only that index mothers had similar pragmatic language violations to mothers of children with Fragile X Syndrome, and both these groups had higher frequency of violations than mothers of typically developing children. Piven et al. (1997) [33] reported higher frequency of pragmatic language violations and speech errors in MPX parents compared to parents of Down's Syndrome children. Additionally, Losh et al. (2008) [34] found a linear trend for both pragmatic language violations and speech errors, reporting MPX parents to score significantly higher than SPX parents who in turn scored significantly higher than parents of children with Down's Syndrome. Ruser et al. (2007) [37] used a modified version of the PRS (PRS-M) and reported index parents to have significantly higher deficits in subscales of emotional expressiveness and awareness of the other, overtalkativeness, and language in comparison to parents of children with Down's Syndrome. Group differences in the communicative factor were not found to be significant; however, index fathers showed significantly increased communication deficits than index mothers. The total PRS-M score revealed significant group differences between index parents and Down's Syndrome parents, with index fathers scoring higher than index mothers.

*Social Responsiveness Scale (SRS)*. The SRS was used as a measure to assess the BAP by two studies in our review (ES range: 0.02–0.90). De la Marche et al. (2012) [38] reported all index fathers (MPX and SPX combined) having a significantly higher total score compared to unaffected adult males; however no statistical differences were found between MPX fathers and SPX fathers and SPX fathers and male controls. In contrast, Schwichtenberg et al. (2010) [39] found that both the MPX and SPX fathers in their sample scored significantly higher than fathers of typically developing children. No differences between mothers in both groups were found.

#### 3.3.2. Other Measures of Personality and Friendships 

Another personality measure used in studies of the BAP is the NEO Personality Inventory (NEO-PI). Two studies show a trend for parents from MPX families scoring significantly higher on the neuroticism subscale in comparison to parents of children from SPX families [34] and parents of DS probands [33, 34] (ES 0.79, *n* = 1). Furthermore, the same two studies assessed quality of friendships using the Friendship Interview (FI), indicating significantly fewer friendships in parents from MPX families in comparison to parents of children from SPX families [34] and parents of Down's Syndrome children [33, 34]. Interestingly, Losh et al. (2008) [34] also found sex differences in the quality of friendships within ASD parents, with fathers from MPX families and SPX families having significantly fewer friendships than mothers from MPX families and SPX families (ES 1.14, *n* = 1).

#### 3.3.3. Reciprocal Social Interaction

Two studies assessed alexithymia (i.e., inability to identify and describe emotions in oneself) as part of the BAP. Szatmari et al. (2008) [9] used the Toronto Alexithymia Scale (TAS-20) as a measure of alexithymia and, despite its three factors (difficulty identifying feelings, difficulty describing feelings, and externally oriented thinking) not reaching significance, the total score confirmed higher frequency of alexithymia in index parents compared to parents of children with Prader Willi syndrome. Using the same scale, however, Berthoz et al. (2013) [40] failed to find a statistically significant difference between index parents and unaffected adults (ES range: 0.14–0.25). Another measure of alexithymia used by Berthoz et al. (2013) [40] was the Bermond-Vorst Alexithymia Questionnaire-B (BVAQ-B); however no significant differences were found between the samples (ES range: 0.02–0.19).

Berthoz et al. (2013) [40] further assessed social anhedonia (i.e., inability to experience pleasure from activities usually found enjoyable), using the revised version of the Social Anhedonia Scale (SAS) (ES 0.25) and found no significant differences between the index parents and unaffected adults. However, Berthoz et al. (2013) [40] found index parents to score significantly higher than unaffected adults on physical anhedonia as measured by the Physical Anhedonia Scale (PAS) (ES 0.33).

#### 3.3.4. Social and Narrative Language

In addition to the PRS, which was specifically designed to assess the deficits in social language as a BAP expression, two other measures have assessed social and narrative language. Di Michele et al. (2007) [8] used Grice's Conversational Maxims task to assess pragmatic conversations and found the index parents performed significantly worse when compared to parents of typically developing children and parents of children with Down Syndrome (ES not available). Landa et al. (1991) [41] used “spontaneous narrative discourse performance” to assess narrative discourse deficits. They reported control adults producing significantly more complete episodes and stories with multiple episodes, and the mean overall quality for the index parents was significantly less than that for the comparison adults (ES range: 0.35–0.73).

#### 3.3.5. Repetitive/Restrictive Behaviors and Interests

Repetitive and restrictive behaviors are a core symptom of autism. The majority of findings in parents of autistic probands corresponding to this domain are covered in the studies that assess the BAP in terms of rigid and perfectionistic personalities. Only one study used an experimental questionnaire designed to examine real-life, nonsocial skills and preferences such as insistence on routines and circumscribed hobbies. Briskman et al. (2001) [42] reported index parents to score significantly higher than parents of boys with dyslexia and typical development (ES range: 0.37–1.11).

### 3.4. Cognitive Domain (Supplementary Table 2)

Most forms of neuropsychological tests involve multiple cognitive functions suggesting that cognitive domains can be related to each other. We have organized the measures for this broad domain under different categories based on the cognitive function which they predominantly assess; however, an overlap may exist. References for the different measures can be found in the studies included in this review and in more specialized text book resources [43].

#### 3.4.1. Intellectual Functioning

Intelligence Quotient (IQ) was measured with different versions of the Wechsler Scales in the studies. Thirteen studies assessed total Verbal IQ (VIQ) (ES range: 0.05–1.28, *n* = 12), with scores for index parents similar to comparison groups in all but one study [44] with higher scores for index parents when compared to parents of Down's Syndrome children. Several VIQ subtests were also independently tested. Three studies used the digit span subtest (some modified it to assess short term memory) (ES range: 0.04–0.67), of which two found better performance in index parents compared to parents with children with Down's Syndrome [44] and parents of children with specific language impairment [23]. Only one study used the Arithmetic subscale and found no significant differences between index parents compared to parents with children with Down's Syndrome [44] (ES: 0.25). Four studies used the vocabulary subtest (ES range: 0.04–0.96) and results were mixed, with one study indicating higher scores for index parents compared to parents of children with Down's Syndrome [44], another indicating a reverse trend with index parents scoring significantly lower than parents of typically developing children [45], and two revealing no significant differences between groups. Four studies assessed the comprehension subtest (ES range: 0.31–0.74), with only one indicating a significant difference with index parents scoring significantly higher than parents of children with Down's Syndrome [44]. Additionally, two studies used the similarities subtest (ES range: 0.13–0.35) with only one reporting a significant difference [44].

Thirteen studies also assessed total Performance IQ (PIQ) (ES range: 0–1.16, *n* = 12), with three studies reporting a significant difference, with index parents performing poorer than parents of children with Down's Syndrome [30, 46] and unaffected adults [10]. One study, however, reported an opposite trend with index fathers performing significantly better than fathers with a child with specific language impairment [47]. Several PIQ subtests were also independently tested. Four studies used the picture completion subtest (ES range: 0.07–0.65); however only two reported significant lower scores for index parents compared to parents of children with Down's Syndrome [30, 46]. Moreover, Folstein et al. (1999) [30] also reported lower scores on the picture arrangement subtest with the same trend of significance (ES range: 0.03–0.26, *n* = 2). Two studies assessed the object assembly subtest (ES range: 0.12–0.62); however only one reported a significant difference with MPX parents scoring lower than parents of Down's Syndrome children [46]. Furthermore, Schmidt et al. (2008) [10] found significantly lower scores on the matrix reasoning subtest in index parents compared to unaffected adults (ES 0.67). Interestingly, none of the five studies assessing the block design subtest (ES range: 0.04–0.43) and one study assessing the digit symbol subtest found significant differences between groups (ES range: 0.17–0.19).

Full Scale IQ (FSIQ) (ES range: 0.05–1.88, *n* = 13) was assessed in fourteen studies in our review with three studies reporting a significant poorer performance in index parents when compared to parents of children with Down's Syndrome [30, 34] and a combined clinical group of parents of children with Down's Syndrome and typical development [48].

Additionally, four studies used Raven's Progressive Matrices to report Nonverbal IQ (NVIQ), with no significant differences found between groups [49–52] (ES range: 0.05–0.57).

#### 3.4.2. Structural Language Abilities

A number of studies assessed structural language abilities using a variety of different measures. Results are divided into specific domains. Receptive language skills were assessed by three studies using two measures. The Peabody Picture Vocabulary Test (PPVT-III) (ES range: 0.33–1.58) was used by two studies with only one study reporting index mothers as having significantly more deficits than mothers of children with autism and language impairment who in turn had more deficits compared to mothers of children with a specific language impairment [47]. Whitehouse et al. (2007) [23] used the Test for Reception of Grammar-2 (TROG-2) to evaluate receptive grammar and reported no differences between groups (ES not available). Schmidt et al. (2008) [10] assessed expressive language using the Expressive Vocabulary Test (EVT) (ES 0.10) and the verbal fluency subtest of the Delis Kaplan Executive Function System (DK-EFS) (ES: 0.16–0.39) reporting no significant differences between index parents and unaffected adults. Additionally, they assessed figurative language using the figurative language subtest from the Test of Language Competence-Expanded Edition (TOLC-E) reporting no significant differences between the two groups (ES: 0.28).

Phonological processing was assessed in five reports using five different tests. Lindgren et al. (2009) [47] used the Comprehensive Test of Phonological Processing (CTOPP) (ES range: 0.02–1.42, *n* = 2), revealing significantly better performance in phonological awareness and the nonword repetition subtests in the index mothers compared to mothers of children with a specific language impairment. In contrast, however, Schmidt et al. (2008) [10] found index parents to perform significantly lower than unaffected adults in the same nonword subtest. Bishop et al. (2004) [53] used a different Nonword Memory Test (ES range: 0.02–0.04) and a Nonsense Passage Reading test (ES range: 0.04–0.42) to assess phonological processing, none indicating significant differences between index parents and parents of typically developing children. However, Whitehouse et al. (2007) [23] did find index parents to perform significantly better than parents of children with specific language impairment in the nonsense words subtest of the NEPSY (a Developmental Neuropsychological Assessment Test Battery) (ES range: 0.04–0.88). In contrast, Plumet et al. (1995) [54] found no significant differences in composite verbal scores when comparing index parents to parents of children with Down's Syndrome using a battery of verbal tasks with an emphasis on orthographic and phonological abilities (ES: 0.22).

Reading skills were assessed by eight studies using seven different measures. Piven and Palmer (1997) [46] used the Rapid Automized Naming (RAN) task and found no differences in the number and letter categories; however, they found significant differences with MPX parents taking longer to complete the task on the color and object categories (ES range: 0.17–0.58). Similarly, Losh et al. (2010) [55] combined the color and object categories and reported index parents taking longer to complete the task when compared with parents of typically developing children (ES not available). The Woodcock-Johnson Psychoeducational Battery-Revised (WJ-R) has several subtests, and no significant differences were found in the broad reading (ES range: 0.48–2.11) and reading skill composite scores [47] (ES range: 0.40–1.84), the word attack subtest [46, 47] (ES range: 0.09–1.35), and letter word subtest [46]. However, Folstein et al. (1999) [30] found a significantly lower reading age and reading grade using the nonsense word reading subtest in index parents compared to parents of children with Down's Syndrome (ES: 0.40). Mothers of children with autism performed better in the dictation (ES range: 0.17–0.99, *n* = 2) and passage comprehension subtests (ES range: 0.45–1.54, *n* = 2) compared to mothers of children with specific language impairment [47]. In contrast, Piven and Palmer (1997) [46] found MPX parents had more difficulties in the passage comprehension subtest when compared with parents of children with Down's Syndrome. Interestingly, no differences were noted in comprehension (ES range: 0.12–0.36) and passage reading subtests (ES range: 0.21–0.36) using the Gray Oral Reading Test (GORT) [30, 44] and the Edinburgh Reading Test (ERT) [44]. Fombonne et al. (1997) [44] also used the National Adult Reading Test (NART) (ES range: 0.20–0.44, *n* = 2) reporting index parents scoring significantly lower than parents of children with Down's Syndrome. However, Baron-Cohen and Hammer (1997) [7] found no significant differences in error scores between index parents and parents of typically developing children. Whitehouse et al. (2007) [23] used the Test of Word Reading Efficiency (ES range: 0.03–0.62) and found index parents performed better than parents of children with specific language impairment on the phonemic decoding efficiency subtest (nonsense words). Finally, Schmidt et al. (2008) [10] found no significant differences in reading difficulties using the Reading History Questionnaire (RHQ) between index parents and unaffected adults (ES: 0.34).

Three studies assessed spelling abilities using two different measures. Whitehouse et al. (2007) [23] found no group differences using a Speeded Dictation task (ES not available). Furthermore, Fombonne et al. (1997) [44] found a superior performance by index parents on the Schonell Spelling Test (SST) (ES range: 0.02–0.13, *n* = 2). Only one study assessed oromotor functioning using the oromotor sequencing subtest of the NEPSY Test Battery (ES range: 0.43–0.54) reporting index families performing better than parents of children with specific language impairment [23].

#### 3.4.3. Social Cognition

In this domain measures assess the ability to process information relating to other people's mental states. Five reports assessed the “Theory of Mind” using different versions of Reading the Mind in the Eyes Test (ES range: 0.03–1.51, *n* = 4). Three studies reported deficits between index parents and comparison groups [7, 48, 56]. In contrast, Gocken et al. (2009) [57] and Tajmirriyahi et al. (2013) [58] found no significant group differences in mental state decoding in the eyes test. Furthermore, Gocken et al. (2009) [57] explored mental state decoding using a faces test and reported no significant differences between index parents and a normative sample (ES: 0.23). Tajmirriyahi et al. (2013) [58], however, used a novel method of Reading the Mind in the Voice Test to reveal significantly higher deficits in mental state decoding in index parents when compared to parents of children with Down's Syndrome and typical development (ES range: 0.63–0.98). Additionally, Di Michele et al. (2007) [8] used False Belief tasks (smarties task, Sally-Anne task, and unexpected transfer test) and found index parents passed fewer false belief tests in comparison to parents of children with Down's Syndrome and typical development (ES not available). Similarly, Gocken et al. (2009) [57] reported poorer performance in index parents compared to a normative sample using the Unexpected Outcomes Test (UOT) (ES: 0.58); however, they did not find a significant difference using the Hinting task (ES: 0.36).

Remarkably, only one study assessed empathy using the Empathy Quotient (EQ) reporting significant impairments in empathy in index fathers compared to unaffected males [52] (ES: 0.11–0.40).

Affect perception was assessed in eight studies using twelve different tests of emotion recognition and labeling. Using the “Bubbles” method with pictures of facial affect, Adolphs et al. (2008) [59] showed no difference in accuracy and reaction time; however, the “BAP+” group used significantly different facial information (eye region and mouth region) in comparison to the “BAP−” group and parents of typically developing children (ES not available). Using the Penn Emotion Recognition Test (ER40), das Neves et al. (2011) [60] reported significantly longer time for correct responses in index parents compared to unaffected adults (ES range: 0.54–1.09). They also reported less accurate responses, identification of female and male faces, and mild and extreme emotions. Bölte and Poustka (2003) [49] showed no significant differences in groups using the Facial Affect Recognition Test (pictures by Ekman and Friesen) (ES range: 0.32–2.06). Similarly, Sucksmith et al. (2013) [52] found no significant differences in accuracy and adjusted response time in index parents compared to unaffected adults using the Karolinska Directed Emotional Faces task (KDEF) (ES range: 0.08–0.30). Kadak et al. (2014) [21] used the Emotion Recognition Test (using photos of facial affect from Ekman and Friesen) and found index parents had impaired recognition of happy, surprised, and neutral faces compared to parents of typically developing children (ES range: 0.05–0.50).

Two studies assessed emotional labeling and matching of facial patterns using three different measures. Using Schematic Line Drawings (ES not available), Palermo et al. (2006) [61] showed impaired labeling for sad, disgust, and overall recognition of facial patterns in index parents compared to parents of typically developing children. In contrast, using the Emotion Matching task (ES: 0.06) and the Emotion Labeling task (ES: 0.19), Smalley and Asarnow (1990) [45] found no significant impairments.

#### 3.4.4. Executive Function

Executive function encompasses abilities that underlie goal directed behavior. This broad domain was split into specific subdomains. Cognitive flexibility was assessed by four studies evaluating set-shifting tasks. Two studies using the intradimensional/extradimensional set-shifting task (IDED) revealed significantly higher rates of learned irrelevance [62] (ES: 0.52), trials to criterion [63] (ES range: 0.69–0.83), and errors to criterion [63] (ES range: 0.64–0.70) in index parents compared to control samples in the extradimensional stage only. However, Bölte and Poustka (2006) [50] used the Wisconsin Card Sorting Test (WCST) (ES range: 0.06–0.18) and the Trail Making Test (TMT, Parts A and B) (ES range: 0.13–0.38) and found no impaired cognitive control between groups. Similarly, Losh et al. (2009) [56] also showed no significant difference in the total time to complete the TMT task between groups.

Five reports assessed planning abilities using two measures. Using the Tower of London (ToL) (ES range: 0.07–0.93, *n* = 2), Hughes et al. (1997) [63] found index parents requiring a significantly increased number of extra moves to complete the task compared to unaffected adults. In contrast, Wong et al. (2006) [62] found no significant group differences in the number of extra moves and rule violations. Three studies used the Tower of Hanoi version (ToH) revealing no significant differences in the total time to complete variable (ES range: 0.01–0.45 *n* = 1) between index parents and a matched clinical sample [50] and nonclinical sample [56], and one study reported significant differences in planning efficiency between index parents and parents of children with Down's Syndrome [46].

One study assessed generativity using the Pattern Meanings test which measures ideational fluency, indicating a significantly impaired overall response generativity in index parents compared to a mixed sample of clinical and nonclinical comparison group [62] (ES: 0.51).

Spatial working memory was assessed by one study using a Visual Search Test, indicating index parents scoring significantly higher between search errors when compared to unaffected adults [63] (ES range: 0.27–0.95). In contrast, however, using the Response to Inhibition and Load (RIL) test, Wong et al. (2006) [62] tested inhibition and its interaction with working memory and found unimpaired reaction times and number of errors in index parents (ES range: 0.04–0.28).

Verbal working memory was assessed using three measures by one study. Using the Stroop Interference Test (ES: 0.2) and a Verbal Fluency Test (letters KAS in Turkish) (ES: 0.26), Gocken et al. (2009) [57] revealed no significant differences between groups. However, they did show impaired accuracy in index parents using the Auditory Consonant Trigrams (ACT) (ES: 0.55).

#### 3.4.5. Local Visual Processing (Central Coherence)

Central coherence is a specific perceptual-cognitive style leading to a local visual processing bias. Five studies assessed disembedding performance using two tests. All five studies used the Embedded Figures Test (EFT) with mixed results. Three out of the five studies found significantly longer response times for index parents [7, 50] and more specifically in index fathers, when compared to control fathers [64] (ES range: 0.01–1.60, *n* = 5). No significant results were reported within the accuracy variable [56, 64] (ES range: 0.11–0.77, *n* = 2); however, De Jonge et al. (2006) [65] reported significantly fewer incorrect responses in index parents when compared to parents of children with Down's Syndrome (ES range: 0.18–0.52). Furthermore, Happé et al. (2001) [64] revealed a similar trend with index parents making fewer errors using the Titchener Circles Illusion test (ES not available).

Mental segmentation ability was assessed with an Unsegmented/Segmented Block Design task (adaptation from the Weschler subtest) in two studies. Happé et al. (2001) [64] found faster response times in index parents in the unsegmented task (ES range: 0.24–0.84, *n* = 1), and, in contrast, Losh et al. (2009) [56] found significantly faster reaction times in the segmented task only (ES range: 0.04–0.63, *n* = 1). Furthermore, De Jonge et al. (2009) [66] showed no group differences in mean number of errors using a Block Design Reconstruction task (patterns by Akshoomoff and Stiles) (ES range: 0.10–0.16).

The sentence completion task was used by two studies to assess global sentence completions revealing significantly increased number of errors in index parents [56, 64] and longer response times in index parents [56].

#### 3.4.6. Visual Processing

Interestingly only one study assessed visual processing using four different measures. Contrast sensitivity was measured using the Vistech Contrast Sensitivity Charts and no significant differences were found between index parents and parents of children with Down's Syndrome [67] (ES: 0.55). Similarly, tasks of motion discrimination (Motion Coherence task (ES: 0.25) and Moving Shape task (ES: 0.17)) and form discrimination (Form Discrimination (Shape) task) (ES: 0.05) revealed no significant differences between the same groups [67].

### 3.5. Other Psychiatric Conditions Domain (Supplementary Table 3)

This domain was assessed in seven reports using nine different measures. Piven et al. (1991) [68] used the Schedule for Affective Disorders and Schizophrenia-Lifetime Version (SADS-L) and found significantly higher scores in the “anxiety” factor when compared to parents of children with Down's Syndrome, and no statistical significance was found for the “major depressive disorder” subscale between the two groups (ES not available). However, using a modified version of the Schedule for Affective Disorders and Schizophrenia-Lifetime Version Modified for the Study of Anxiety Disorders, Revised (SADS-LA-R), Piven and Palmer (1999) [69] did find significantly higher frequency of “major depressive disorder” in index parents in addition to the “social phobia” factor.

Micali et al. (2004) [70] devised a parental questionnaire and validated their results from consented medical records from GPs and found a significant trend towards higher prevalence of “depression” and “anxiety” in index parents. Using the Symptom Checklist-90-Revised (SCL-90-R), Bölte et al. (2007) [51] found significantly increased frequency in index parents in four of the nine subscales (depression, hostility, phobic anxiety, and paranoid ideation) (ES range: 0–1.33). Additionally, Bölte et al. (2007) [51] also assessed personality style and disorder using the Personality Style and Disorder Inventory (PSSI) and reported significantly higher rates in index parents in five out of fourteen factors (reserved/schizoid, self-critical/insecure, critical/negativistic, spontaneous/borderline, and quiet/depressive) (ES range: 0.02–1.18).

Gocken et al. (2009) [57] assessed depression and anxiety factors using the Brief Psychiatric Rating Scale (BPRS) between index parents and a normative comparison group and only found a statistically significant difference in the depression factor with index parents scoring higher (ES range: 0.29–0.44). Similarly, Ingersoll et al. (2011) [25] assessed depressed mood using the Centre for Epidemiological Studies-Depression Scales (CESD) and showed index mothers as having increased rates of depression when compared to a normative sample of mothers (ES: 0.35). Interestingly, Berthoz et al. (2013) [40] reported no significant differences in levels of depressive mood using the Beck Depression Inventory (BDI) (ES: 0.50) and no significant differences were found in anxiety levels using the state (ES: 0.19) and trait portions (ES: 1.24) of State-Trait Anxiety Inventory Form Y (STAI-Y) [40].

## 4. Discussion

This systematic review aimed to assess the evidence of behavioral, cognitive, and psychiatric profiles of the BAP in unaffected biological parents of autistic probands by synthesizing the evidence from 60 studies meeting a priori search criteria. Results are discussed according to the following criteria: (i) the number of studies that indicate significant impairments in each domain and subdomain; (ii) quantitative criteria using effect sizes; and (iii) the possible emerging themes across studies.  Table 2 represents a summary of all measures used by studies meeting our search criteria.

### 4.1. Summary of Findings

Findings emerging from this review are discussed according to each domain. Within the sociobehavioral domain, eight measures that directly assess the BAP expression in unaffected parents showed substantial deficits in the domain of social and communication skills (AQ, 7/10 studies; BPASS, 1 study; CC-A, 1 study; FHI/FHS, 2/2 studies; SRS, 2/2 studies), rigid and perfectionistic (BAPQ, 2/2 studies; MPAS-R, 3/3 studies) and aloof (BAPQ, 2/2; MPAS-R, 3/4 studies) personality traits, and pragmatic language difficulties (BAPQ, 2/2 studies; PRS, 4/4 studies) related to the core deficit in autism and are reported consistently across most studies. Moreover, additional deficits in social and narrative language have been highlighted using measures of spontaneous narrative discourse [36] and Grice's Conversational Maxims task [8]. Available evidence also points to index parents establishing fewer friendships (FI, 2/2 studies) and an elevated frequency of neuroticism (NEO-PI, 2/2 studies). Despite being a core domain of a clinical diagnosis for autism, the majority of findings in parents of autistic probands corresponding to restricted and repetitive behaviors and interests are covered in the studies that assess the BAP in terms of rigid and perfectionistic personality styles. Only one study used an experimental questionnaire designed to examine real-life nonsocial skills and preferences such as insistence on routines and circumscribed hobbies [42].

Within the sociobehavioral domain, reciprocal social interaction is probably the least studied subdomain in parents of autistic probands. As such, findings from alexithymia (TAS-20, 1/2 studies; BVAQ-B, 1 study with no significance found) and physical (PAS, 1/1 study) and social anhedonia (SAS, 1 study with no significance found) are modest and require further studies to explore these traits. Thus, we agree with previous reviews [12–14] indicating that mild social/communication deficits, rigid/aloof personality traits, and pragmatic language difficulties may be the most useful social behavioral candidate endophenotype traits as they meet all the established criteria [11]; however, effect sizes throughout this domain varied considerably.

At the cognitive level, a remarkable finding is the discrepancies found in intellectual functioning of parents of autistic probands compared to parents of children with and without a clinical diagnosis. One of thirteen studies revealed significantly higher VIQ scores when compared to a clinical sample of parents of a child with Down's Syndrome [44]. Three of thirteen studies assessing PIQ reached a similar significant trend when compared to parents with a Down's Syndrome child [30, 46] and unaffected adults [10]. Total PIQ scores were significantly higher in index parents when compared to parents with a child with specific language impairment [47]. Only two of twelve reports reached a significant deficit in FSIQ when index parents were compared to parents of children with Down's Syndrome [30] and when compared to a combined sample of parents of a child with Down's Syndrome and of typical development. However, it is noteworthy that scores for all parents were well within the average range in all studies. Thus there is limited evidence for the role of intellectual functioning as an endophenotype for autism with no clear clinical significance.

Several measures were used to assess the structural language abilities within the cognitive domain. Interestingly, no significant differences were found in the expressive language (TROG-2, 1 study with no significance found; EVT, 1 study with no significance found; DK-EFS verbal fluency subtest, 1 study with no significance found) and figurative language categories (TOLCE-E figurative language subtest, 1 study with no significance found). Lindgren et al. (2009) [47] found index parents to perform better than parents with a child with a specific language impairment on measures assessing receptive language (PPVT-III, 1/2 studies; TROG-2, 1 study with no significance found) refuting the hypothesis that families with autism and specific language impairment do not share similar genetic loading for language.

In phonological awareness, findings are mixed with studies only reporting few deficits in nonsense word/passage reading tests (2/3 studies) with index parents performing better than parents with a specific language impairment child [23] and parents of children with Down's Syndrome [30]. Using the RAN measure for reading skills, two studies reported faster times to complete the color and object only tasks in index parents when compared to parents of children with Down's Syndrome [46] and parents of typically developing children [55]. This may have relevance with regard to perceptual load in autism. However, no significant differences were found in the rapid naming subtest of the CTOPP [47].

Findings from the social cognition domain including mental state decoding, affect perception, emotion recognition, and labeling in the BAP also report mixed and conflicting results. Remarkably only one studied assessed empathy warranting further research in this subdomain.

Evidence from the broad domain of executive function in the BAP is also inconsistent but the few studies that have found impairments did not appropriately match experimental and control groups for IQ are worth noting (e.g., [63]).

Similarly, findings from studies assessing performance on tests where local visual processing is an advantage (central coherence) were mixed in studies of the BAP. Conflicting results in the disembedding performance were noted (EFT, 4/8 studies; Titchener Circles Illusion, 1 study) as well as mental segmentation abilities (Unsegmented Block Design task, 1/2 studies; Segmented Block Design task, 1/2 studies; Block Design Reconstruction task, 1 study with no significance found). Two studies, however, indicate higher frequency of errors and response times in index parents during a global sentence completion task (sentence completion task, 2/2 studies). Nonetheless, this area of cognition in the BAP also warrants further research.

Lastly, a number of studies have documented higher rates of depression (in 5/7 measures), anxiety (in 2/6 measures), and social phobia/social phobic anxiety (in 4/6 measures) in parents of children with autism compared to normative samples (e.g., [57]) and a clinical sample (e.g., [51]). We also note depression and anxiety to be more prevalent (2/6 studies) in mothers of children with autism. Ingersoll et al. (2011) [25] reported increased depressed mood in index mothers when compared to mothers of typically developing children, with similar findings from Micali et al. (2004) [70]. Although one can assume that having a child with a disability can affect mood and anxiety levels, many studies indicate an onset of these conditions before the birth of the child with autism, suggesting that the stress of caring for a child with a disability did not cause the symptoms. Findings from our review revealed moderate to high magnitude of effect; thus, depression and anxiety may have a genetic link with autism, supporting findings from a previous meta-analysis of psychiatric disorders in parents of children with autism [71].

**Figure 2:**
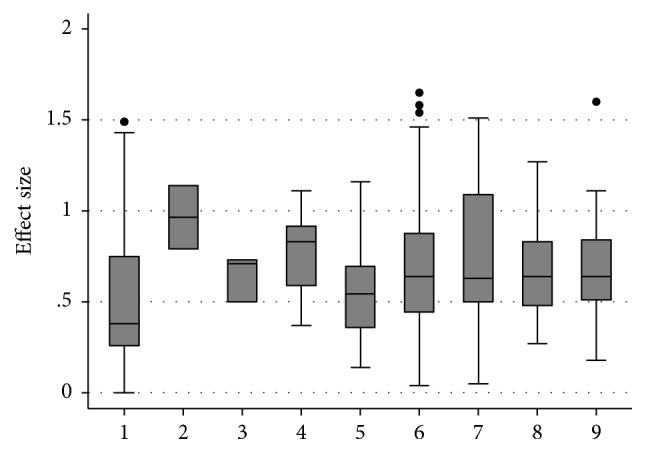
Boxplot reflecting effect size ranges for the sociobehavioral and cognitive domains. 1 = BAP expression. 2 = other measures of personality and friendships. 3 = social and narrative language. 4 = repetitive, restrictive behaviors, and interests. 5 = general intellectual functioning. 6 = structural language abilities. 7 = social cognition. 8 = executive function. 9 = local visual processing (central coherence).

Figure 2 displays the boxplots reflecting effect size ranges for the sociobehavioral and cognitive domains and subdomains. It was not possible to include effect size ranges for the domain of other psychiatric conditions as depression and anxiety could not be divided into separate subdomains due to the measures used in the studies. The reciprocal social interaction subdomain was omitted as there was only one effect size available for one significant finding. Similarly, the visual processing subdomain was also omitted as findings were not significant.

### 4.2. Emerging Themes

A number of studies reviewed suggest that subclinical autistic traits aggregate in MPX families and occur less frequently in SPX families [28, 34]. For instance decreased number and intensity of BAP traits observed in parents of SPX in comparison to MPX provide behavioral evidence consistent with findings of increased de novo, noninherited genetic events in SPX families (e.g., [72]). Losh et al. (2008) [34] suggest that the BAP gradation expression across family types is consistent with increasing genetic liability to autism.

A male bias is a well-documented feature in autism [73]. Findings from our review also indicate few sex differences, indicating this male bias [37–39]. However, despite this and the clear sex bias in autism, many studies do not suggest sex differences for most BAP features (e.g., [74]).

Furthermore, our findings indicate that the majority of the studies reviewed were conducted in Western countries. There were too few studies from non-Western countries to make any meaningful comparisons. Further cross-cultural research is required to understand the endophenotypes of autism within different cultural and geographical settings in order to tackle this geographical distribution bias.

### 4.3. Measure Quality

It is clear from this review that a large number of measures have been utilized to assess the BAP in relation to different domains and the constructs analyzed are heterogeneous. However it should be noted that the current review does not assess in depth whether the BAP measures are valid or reliable in measuring BAP. Domain wise, in many cases the same measures have been used by other studies. We discuss whether results for each measure in the same domain show the same magnitude and are in the same direction.

For instance, Davidson et al. (2014) [75] reported that frequency of BAP traits varies significantly depending upon the measure utilized, highlighting the need for a different approach that utilizes multiple informants and relies on the assessment of distinct BAP traits.

### 4.4. Methodological Limitations of Studies

Any discordant findings in the studies reviewed may be partly explained by methodological differences between studies. Sample size and choice of comparison group play an important role in the outcome of results. Six studies enrolled 30 or less index parents. Thus, relatively small sample sizes may lead to false negative results and/or limit the power to detect the BAP in the three domains.

Studies vary in their choice of a comparison group with some relying on the convenience of clinic-based samples where selection biases may lead to distorted results and others emphasizing the use of population based samples. For example, parents of children with Down Syndrome were frequently used, but these parents are likely to be older and possibly of different socioeconomic status. Few studies matched index parents to control groups on intellectual functioning, age, and socioeconomic basis, thus making it difficult to assimilate if differences on specific cognitive tasks represent a specific impairment in functioning or are attributable to differences in demographic data.

### 4.5. Limitations and Future Directions

In addition to the limitation outlined above, there are other limitations. Given that nine additional studies were found through a manual search after the initial search, it is possible that other studies were not ascertained by our search terms. To address this limitation, future research may also consider additional search terms beyond those used here.

This review aimed to identify endophenotypes in behavioral, cognitive, and psychiatric domains independently, and as such we did not assess associations between the BAP features across different domains. Losh et al. (2009) [56] suggest that it is likely that specific BAP traits cosegregate with performance in other domains. For instance, parents displaying rigid/perfectionistic personality traits could perform differently on tasks requiring cognitive flexibility. Additionally, most studies meeting our search criteria assessed only one or two domains, rendering it difficult to establish whether an endophenotypic overlap, if any, exists.

Future reviews should also include studies that examine neuroanatomical and neurofunctional correlates of the BAP. These are essential in furthering our understanding of the neural correlates of the behavioral, cognitive, and psychiatric aspects of autism.

More sophisticated research of the endophenotypes of parents of children with autism may help develop better measures of evaluation of the BAP. Future studies should use a more comprehensive and quantitative framework using more robust measures to detect subtle subclinical autistic traits in the BAP in cross-cultural settings. To the best of our knowledge, no study assessing the endophenotypic profile of autism in Africa has been published yet. Such research by our team is underway.

### 4.6. Conclusions

In summary, the current review increases our understanding of the BAP and extends the findings of previous reviews [13, 14]. It also supplements a systematic review [12] and a meta-analysis [71] with a broader scope. However, findings should be interpreted with caution because of the small number of studies in such heterogeneously broad domains and methodological limitations.

The assessment of the BAP profile in parents of autistic probands allows us to have a better insight into the varying underlying genetic mechanisms in autism. The behavioral, cognitive, and psychiatric endophenotypes in parents of autistic probands are still not clarified; however, evidence points towards mild social/communication deficits, rigid/aloof personality traits, and pragmatic language difficulties as the most useful social behavioral candidate endophenotype traits. The existence of some deficits in the cognitive domain does suggest familial vulnerability for autism; however, more research is required to elucidate these findings within this domain. Furthermore, increased depressed mood and anxiety can also be useful markers of vulnerability.

## Supplementary Material

Supplementary Table 1 reviews the socio-behavioral endophenotypes in all studies of parents of autistic probands meeting our search criteria. The domains reviewed in this matrix are: BAP expression, other measures of personality and friendships, reciprocal social interaction, social and narrative language and repetitive, restrictive behaviors and interests.
Supplementary Table 2 reviews the cognitive endophenotypes in all studies of parents of autistic probands meeting our search criteria. The domains reviewed in this matrix are: general intellectual functioning, structural language abilities, social cognition, executive function, central coherence (local visual processing) and visual processing.
Supplementary Table 3 reviews other psychiatric endophenotypes in all studies of parents of autistic probands meeting our search criteria. The domains reviewed in this matrix are: depression and anxiety.

## Figures and Tables

**Table 1:** Description of search strategy.

Search element	EMBASE	MEDLINE	PsycINFO	PsycEXTRA	Global Health
Exposure	Thesaurus terms explored: Autis^*∗*^				

Keywords	Endophenotyp^*∗*^ OR Phenotyp^*∗*^				

Outcome	*Thesaurus terms explored* Behavior Language Social interaction Repetitive Restrictive Cognitive Executive function Central coherence Theory of mind Social cognition Visual Attention Depression anxiety	*Thesaurus terms explored* Behavior Language Social interaction Repetitive Restrictive Cognitive Executive function Central coherence Theory of mind Social cognition Visual Attention Depression anxiety	*Thesaurus terms explored* Behavior Language Social interaction Repetitive Restrictive Cognitive Executive function Central coherence Theory of mind Social cognition Visual Attention Depression anxiety	*Thesaurus terms explored* Behavior Language Social interaction Repetitive Restrictive Cognitive Executive function Central coherence Theory of mind Social cognition Visual Attention Depression anxiety	*Thesaurus terms explored* Behavior Language Social interaction Repetitive Restrictive Cognitive Executive function Central coherence Theory of mind Social cognition Visual Attention Depression anxiety

Population	Parent^*∗*^ OR Relative^*∗*^ OR Famil^*∗*^				

Language	Any				

**Table 2:** Summary of the frequency of all measures used by studies meeting our search criteria and effect size ranges for each domain.

		Frequency
Sociobehavioral category		
BAP expression (ES range: 0.01–1.49)		
Autism Spectrum Quotient (AQ)		10
Broader Autism Phenotype Questionnaire (BAPQ)		2
Broader Phenotype Autism Spectrum Scale (BPASS)		1
Communication Checklist-Adult (CC-A)		1
Family History Interview/Family History Schedule (FHI/FHS)		3
Modified Personality Assessment Schedule-Revised (MPAS-R)		4
Pragmatic Rating Scale (PRS)		4
Social Responsiveness Scale (SRS)		2
Other measures of personality and friendships (ES range: 0.79–1.14)		
The Friendship Interview (FI)		2
The Neo Personality Interview (NEO-PI)		2
Reciprocal social interaction (ES: 0.33)		
*Alexithymia*		
Toronto Alexithymia Scale (TAS-20)		2
Bermond-Vorst Alexithymia Questionnaire-B (BVAQ-B)		1
*Anhedonia*		
Revised Social Anhedonia Scale (SAS)		1
Physical Anhedonia Scale (PAS)		1
Social and narrative language (ES: 0.50–0.73)		
Grice's Conversational Maxims task		1
Spontaneous Narrative Language		1
Repetitive, restrictive behaviors & interests (ES: 0.37–1.11)		
*Everyday Preferences & Abilities*		
Real Life Skills & Preferences		1

Cognitive category		
General intellectual functioning (ES range: 0.14–1.16)		
Wechsler Scales		19
Raven's Progressive Matrices (RPM)		4
Structural language abilities (ES range: 0.04–1.65)		
*Receptive language*		
Peabody Picture Vocabulary Test (PPVT-III)		2
Test for Reception of Grammar-2 (TROG-2)		1
*Expressive language*		
Expressive Vocabulary Test (EVT)		1
Verbal Fluency Subtest-Delis Kaplan Executive Function System (DK-EFS)		1
*Figurative language*		
Figurative Language Subtest-Test of Language Competence-Expanded (TOLC-E)		1
*Phonological awareness*		
Comprehensive Test of Phonological Processing (CTOPP)		2
Nonword Memory Test		1
Nonsense Passage Reading Test		1
Nonsense Words Subtest-NEPSY Test Battery		1
Battery of Verbal tasks (including orthographic & phonological abilities)		1
*Reading abilities*		
Rapid Automized Naming (RAN)		2
Woodcock-Johnson Psychoeducational Battery-Revised (WJ-R)		3
Gray Oral Reading Test (GORT)		2
Edinburgh Reading Test (ERT)		1
National Adult Reading Test (NART)		2
Test of Word Reading Efficiency		1
Reading History Questionnaire (RHQ)		1
*Spelling abilities*		
Schonell Spelling Test (SST)		1
Speeded Dictation task		2
*Oromotor functioning*		
Oromotor Sequencing Subtest-NEPSY Test Battery		1
Social cognition (ES range: 0.05–1.51)		
*Theory of Mind*		
Reading the Mind in the Eyes Test (different versions)		5
The Faces Test		1
Reading the Mind in the Voice Test		1
False Belief tasks (Smarties task; Sally-Anne task; unexpected transfer test)		1
Unexpected Outcomes Test (UOT)		1
The Hinting task		1
*Empathy*		
Empathy Quotient (EQ)		1
*Affect perception/emotion recognition*		
Pictures of facial affect, “Bubbles” method		1
Penn Emotion Recognition Test (ER40)		1
Facial Affect Recognition Test		1
Emotion Recognition Test		1
Karolinska Directed Emotional Faces task (KDEF)		1
Point Light Basic Emotions task		1
Trustworthiness of Faces task		1
The Morphed Faces task		1
The Movie Still task		1
Schematic Line Drawings task		1
Emotion Matching task		1
Emotion Labeling task		1
Executive function (ES range: 0.27–1.27)		
*Set-shifting*		
intradimensional-extradimensional Set-Shifting task (IDED)		2
Wisconsin Card Sorting Test (WCST)		1
Trail Making Test (A & B)		2
*Planning*		
Tower of London (ToL)		2
Tower of Hanoi (ToH)		3
*Generativity/ideational fluency*		
Pattern Meanings		1
*Spatial working memory/inhibition*		
Visual Search Test		1
The Delayed Oculomotor task		1
Response Inhibition & Load (RIL)		1
*Verbal working memory*		
Auditory Consonant Trigrams (ACT)		1
Verbal Fluency Test		1
Stroop Interference Test		1
Central coherence (local visual processing) (ES range: 0.18–1.60)		
*Disembedding performance*		
Embedded Figures Test (EFT)		5
Titchener Circles Illusion		1
*Mental segmentation ability*		
Unsegmented Block Design task (adapted from Wechsler Scales)		2
Segmented Block Design task (adapted from Wechsler Scales)		2
Block Design task (Wechsler scales)		2
Block Design Reconstruction task		1
*Attentional engagement*		
Detection task		1
*Global sentence completions*		
Sentence completion task		2
Visual processing (ES not available)		
*Contrast sensitivity*		
Vistech Contrast Sensitivity Charts		1
*Motion discrimination*		
Motion Coherence task		1
Moving Shape task		1
*Form discrimination*		
Form Discrimination (Shape) task		1
Other psychiatric conditions category (depression and anxiety) (ES range: 0–1.33)		
Brief Psychiatric Rating Scale (BPRS)		1
Personality Style & Disorder Inventory (PSSI)		1
Symptom Checklist 90-Revised (SCL-90-R)		1
Schedule for Affective Disorders and Schizophrenia-Lifetime Version (SADS-L)		1
Schedule for Affective Disorders and Schizophrenia-Lifetime Version Modified for the Study of Anxiety Disorders-Revised (SADS-LA-R)		1
Parental questionnaire		1
The Centre for Epidemiological Studies-Depression Scales (CESD)		1
Beck Depression Inventory		1
State-Trait Anxiety Inventory Form Y (STAI-Y)		1
