# Supplementary material for: Investigating the Evidence of Behavioral, Cognitive, and Psychiatric Endophenotypes in Autism: A Systematic Review
Source: Autism Res Treat. 2017 Jul 5;2017:6346912. doi: 10.1155/2017/6346912 (PMC5516739; doi:10.1155/2017/6346912)
Supplement: Supplementary file 1 — Supplementary Table 1 reviews the socio-behavioral endophenotypes in all studies of parents of autistic probands meeting our search criteria. The domains reviewed in this matrix are: BAP expression, other measures of personality and friendships, reciprocal social interaction, social and narrative language and repetitive, restrictive behaviors and interests. Supplementary Table 2 reviews the cognitive endophenotypes in all studies of parents of autistic probands meeting our search criteria. The domains reviewed in this matrix are: general intellectual functioning, structural language abilities, social cognition, executive function, central coherence (local visual processing) and visual processing. Supplementary Table 3 reviews other psychiatric endophenotypes in all studies of parents of autistic probands meeting our search criteria. The domains reviewed in this matrix are: depression and anxiety. [file 6346912.f1.pdf]

Supplementary Table 1. Socio-Behavioral Endophenotype Matrix – Review of studies of parents of autistic probands.

Domain	Method / Measure	Factors / Subscales	Country	Study	ASD Parent Group Characteristics	Control Group Characteristics	Key Findings in relation to Proband Diagnosis	
							P value	Effect Size (*d*)
**BAP Expression *(Measures designed specifically to assess BAP)***	**Autism Spectrum Quotient (AQ)** *Self-report Questionnaire*	Social Skills	UK	Berthoz et al (2013) [40]	ASD-P n = 87 (28%Fa)	UA n = 47 (62%M)	**n.s.**	**ASD-P vs. UA 0.36**
			Australia	Bishop et al (2004a) [20]	ASD-P n = 111 (65Mo/46Fa)	N-P n = 85 (48Mo/37Fa)	**ASD-P > N-P****	**ASD-Mo vs. N-Mo 0.22**<br>**ASD-Fa vs. N-Fa 0.60**
			Turkey	Kadak et al (2014) [21]	ASD-P n=72 (36Mo/36Fa)	TD-P n=38 (19Mo/19Fa)	**ASD-P > TD-P****	**ASD-P vs. TD-P 0.5**
			Turkey	Kose et al (2013) [18]	ASD-P n = 100 (53Mo/47Fa)	TD-P n = 100 (52Mo/48Fa)	**ASD-P > TD-P****	**ASD-Fa vs. TD-Fa 0.43**<br>**ASD-Mo vs. TD-Mo 0.30**
			Italy	Ruta et al (2012) [17]	ASD-P n= 245 (130Mo/115Fa)	TD-P n = 300 (150Mo/150Fa)	**ASD-P > TD-P****	**ASD-Fa vs. TD-Fa 0.25**<br>**ASD-Mo vs. TD-Mo 0.24**
			Netherlands	Scheeren & Stauder (2008) [24]	ASD-P n= 25 (12Mo/13Fa)	TD-P n= 25 (12Mo/13Fa)	**n.s**	
			UK	Wheelwright et al (2010) [22]	ASD-P n= 2000 (1429Mo/571Fa)	TD-P n= 1007 (658Mo/349Fa)	**ASD-P > TD-P*****	**ASD-Fa vs. TD-Fa 0.33**<br>**ASD-Mo vs. TD-Mo 0.46**
			UK	Whitehouse et al (2007) [23]	ASD-P n = 30 (20Mo/10Fa)	SLI-P n= 25	**n.s.**	**ASD-P vs. SLI-P 0.67**
		Attention Switching	UK	Berthoz et al (2013) [40]	ASD-P n = 87 (28%Fa)	UA n = 47 (62%M)	**n.s.**	**ASD-P vs. UA 0.14**
			Australia	Bishop et al (2004a) [20]	ASD-P n = 111 (65Mo/46Fa)	N-P n = 85 (48Mo/37Fa)	**n.s.**	**ASD-Mo vs. N-Mo 0.13**<br>**ASD-Fa vs. N-Fa 0.19**
			Turkey	Kadak et al (2014) [21]	ASD-P n=72 (36Mo/36Fa)	TD-P n=38 (19Mo/19Fa)	**n.s.**	**ASD-P vs. TD-P 0.09**
			Turkey	Kose et al (2013) [18]	ASD-P n = 100 (53Mo/47Fa)	TD-P n = 100 (52Mo/48Fa)	**n.s.**	**ASD-Fa vs. TD-Fa 0.33**<br>**ASD-Mo vs. TD-Mo 0.15**
			Italy	Ruta et al (2012) [17]	ASD-P n= 245 (130Mo/115Fa)	TD-P n = 300 (150Mo/150Fa)	**n.s.**	**ASD-Fa vs. TD-Fa 0.13**<br>**ASD-Mo vs. TD-Mo 0.03**

	Netherlands	Scheeren & Stauder (2008) [24]	ASD-P n= 25 (12Mo/13Fa)	TD-P n= 25 (12Mo/13Fa)	**n.s**	
	UK	Wheelwright et al (2010) [22]	ASD-P n= 2000 (1429Mo/571Fa)	TD-P n= 1007 (658Mo/349Fa)	**ASD-P > TD-P*** **	**ASD-Fa vs. TD-Fa 0.12**<br>**ASD-Mo vs. TD-Mo 0.38**
	UK	Whitehouse et al (2007) [23]	ASD-P n = 30 (20Mo/10Fa)	SLI-P n= 25	**ASD-P > SLI-P***	**ASD-P vs. SLI-P 0.67**
Attention to Detail	UK	Berthoz et al (2013) [40]	ASD-P n = 87 (28%Fa)	UA n = 47 (62%M)	**n.s.**	**ASD-P vs. UA 0.37**
	Australia	Bishop et al (2004a) [20]	ASD-P n = 111 (65Mo/46Fa)	N-P n = 85 (48Mo/37Fa)	**n.s.**	**ASD-Mo vs. N-Mo 0.29**<br>**ASD-Fa vs. N-Fa 0.12**
	Turkey	Kadak et al (2014) [21]	ASD-P n=72 (36Mo/36Fa)	TD-P n=38 (19Mo/19Fa)	**n.s.**	**ASD-P vs. TD-P 0.22**
	Turkey	Kose et al (2013) [18]	ASD-P n = 100 (53Mo/47Fa)	TD-P n = 100 (52Mo/48Fa)	**n.s.**	**ASD-Fa vs. TD-Fa 0.04**<br>**ASD-Mo vs. TD-Mo 0.14**
	Italy	Ruta et al (2012) [17]	ASD-P n= 245 (130Mo/115Fa)	TD-P n = 300 (150Mo/150Fa)	**n.s.**	**ASD-Fa vs. TD-Fa 0.04**<br>**ASD-Mo vs. TD-Mo 0.03**
	Netherlands	Scheeren & Stauder (2008) [24]	ASD-P n= 25 (12Mo/13Fa)	TD-P n= 25 (12Mo/13Fa)	**TD-Mo > ASD-Mo***	
	UK	Wheelwright et al (2010) [22]	ASD-P n= 2000 (1429Mo/571Fa)	TD-P n= 1007 (658Mo/349Fa)	**n.s.**	**ASD-Fa vs. TD-Fa 0.13**<br>**ASD-Mo vs. TD-Mo 0.13**
	UK	Whitehouse et al (2007) [23]	ASD-P n = 30 (20Mo/10Fa)	SLI-P n= 25	**n.s.**	**ASD-P vs. SLI-P 0.13**
Communication	UK	Berthoz et al (2013) [40]	ASD-P n = 87 (28%Fa)	UA n = 47 (62%M)	**n.s.**	**ASD-P vs. UA 0.13**
	Australia	Bishop et al (2004a) [20]	ASD-P n = 111 (65Mo/46Fa)	N-P n = 85 (48Mo/37Fa)	**ASD-P > N-P****	**ASD-Mo vs. N-Mo 0.19**<br>**ASD-Fa vs. N-Fa 0.52**
	Turkey	Kadak et al (2014) [21]	ASD-P n=72 (36Mo/36Fa)	TD-P n=38 (19Mo/19Fa)	**n.s.**	**ASD-P vs. TD-P 0.32**

	Turkey	Kose et al (2013) [18]	ASD-P n = 100 (53Mo/47Fa)	TD-P n = 100 (52Mo/48Fa)	**ASD-P > TD-P****	**ASD-Fa vs. TD-Fa 0.20**<br>**ASD-Mo vs. TD-Mo 0.62**
	Italy	Ruta et al (2012) [17]	ASD-P n= 245 (130Mo/115Fa)	TD-P n = 300 (150Mo/150Fa)	**ASD-P > TD-P****	**ASD-Fa vs. TD-Fa 0.02**<br>**ASD-Mo vs. TD-Mo 0.01**
	Netherlands	Scheeren & Stauder (2008) [24]	ASD-P n= 25 (12Mo/13Fa)	TD-P n= 25 (12Mo/13Fa)	**n.s**	
	UK	Wheelwright et al (2010) [22]	ASD-P n= 2000 (1429Mo/571Fa)	TD-P n= 1007 (658Mo/349Fa)	**ASD-P > TD-P*****	**ASD-Fa vs. TD-Fa 0.22**<br>**ASD-Mo vs. TD-Mo 0.41**
	UK	Whitehouse et al (2007) [23]	ASD-P n = 30 (20Mo/10Fa)	SLI-P n= 25	**ASD-P > SLI-P***	**ASD-P vs. SLI-P 0.63**
Imagination	UK	Berthoz et al (2013) [40]	ASD-P n = 87 (28%Fa)	UA n = 47 (62%M)	**n.s.**	**ASD-P vs. UA 0.10**
	Australia	Bishop et al (2004a) [20]	ASD-P n = 111 (65Mo/46Fa)	N-P n = 85 (48Mo/37Fa)	**n.s.**	**ASD-Mo vs. N-Mo 0.07**<br>**ASD-Fa vs. N-Fa 0.19**
	Turkey	Kadak et al (2014) [21]	ASD-P n=72 (36Mo/36Fa)	TD-P n=38 (19Mo/19Fa)	**n.s.**	**ASD-P vs. TD-P 0.02**
	Turkey	Kose et al (2013) [18]	ASD-P n = 100 (53Mo/47Fa)	TD-P n = 100 (52Mo/48Fa)	**n.s.**	**ASD-Fa vs. TD-Fa 0.03**<br>**ASD-Mo vs. TD-Mo 0.07**
	Italy	Ruta et al (2012) [17]	ASD-P n= 245 (130Mo/115Fa)	TD-P n = 300 (150Mo/150Fa)	**n.s.**	**ASD-Fa vs. TD-Fa 0.06**<br>**ASD-Mo vs. TD-Mo 0.57**
	Netherlands	Scheeren & Stauder (2008) [24]	ASD-P n= 25 (12Mo/13Fa)	TD-P n= 25 (12Mo/13Fa)	**n.s**	
	UK	Wheelwright et al (2010) [22]	ASD-P n= 2000 (1429Mo/571Fa)	TD-P n= 1007 (658Mo/349Fa)	**ASD-P > TD-P*****	**ASD-Fa vs. TD-Fa 0.04**<br>**ASD-Mo vs. TD-Mo 0.29**
	UK	Whitehouse et al (2007) [23]	ASD-P n = 30 (20Mo/10Fa)	SLI-P n= 25	**n.s.**	**ASD-P vs. SLI-P 0.41**
AQ Total Score	UK	Berthoz et al (2013) [39]	ASD-P n = 87 (28%Fa)	UA n = 47 (62%M)	**n.s.**	**ASD-P vs. UA 0.06**

		Turkey	Kadak et al (2014) [21]	ASD-P n=72 (36Mo/36Fa)	TD-P n=38 (19Mo/19Fa)	**n.s.**	**ASD-P vs. TD-P 0.39**
		Turkey	Kose et al (2013) [18]	ASD-P n = 100 (53Mo/47Fa)	TD-P n = 100 (52Mo/48Fa)	**ASD-P > TD-P***	**ASD-Fa vs. TD-Fa 0.34**<br>**ASD-Mo vs. TD-Mo 0.27**
		Italy	Ruta et al (2012) [17]	ASD-P n= 245 (130Mo/115Fa)	TD-P n = 300 (150Mo/150Fa)	**ASD-P > TD-P****	**ASD-Fa vs. TD-Fa 0.30**<br>**ASD-Mo vs. TD-Mo 0.29**
		Netherlands	Scheeren & Stauder (2008) [24]	ASD-P n= 25 (12Mo/13Fa)	TD-P n= 25 (12Mo/13Fa)	**n.s**	**ASD-Fa vs. TD-Fa 0.30**<br>**ASD-Mo vs. TD-Mo 0.73**
		UK	Wheelwright et al (2010) [22]	ASD-P n= 2000 (1429Mo/571Fa)	TD-P n= 1007 (658Mo/349Fa)	**ASD-P > TD-P*****	**ASD-Fa vs. TD-Fa 0.17**<br>**ASD-Mo vs. TD-Mo 0.38**
		UK	Whitehouse et al (2007) [23]	ASD-P n = 30 (20Mo/10Fa)	SLI-P n= 25	**ASD-P > SLI-P***	**ASD-P vs. SLI-P 0.63**
	AQ Social/Communication score combined	USA	Ingersoll et al (2011) [25]	ASD-Mo n = 71 (Only Mo)	N-Mo n = 94 (Only Mo)	**ASD-Mo > N-Mo***	**ASD-Mo vs. N-Mo 0.33**
	F1 (communication & socialization)	France	Robel et al (2014) [19]	ASD-P n = 66 (35Mo/31Fa)	TD-P n = 127 (67Mo/60Fa)	**ASD-P > TD-P***	**ASD-P vs. TD-P 1.34**
	F2 (imagination & rigidity)	France	Robel et al (2014) [19]	ASD-P n = 66 (35Mo/31Fa)	TD-P n = 127 (67Mo/60Fa)	**n.s.**	**ASD-P vs. TD-P 0.24**
	Global score (F1 & F2 combined)	France	Robel et al (2014) [19]	ASD-P n = 66 (35Mo/31Fa)	TD-P n = 127 (67Mo/60Fa)	**ASD-P > TD-P***	**ASD-P vs. TD-P 0.76**
**Broader Autism Phenotype Questionnaire (BAPQ)**	Aloof	USA	Hurley et al (2007) [26]	ASD-P = 86 (40Mo/46Fa)<br>*BAP(+) n = 27*<br>*BAP(-) n = 59*	N-P = 64 (32Mo/32Fa)	**BAP(+) > BAP(-), N-P*****	**BAP(+) vs. BAP(-) 1.49**<br>**BAP(+) vs. N-P 1.30**
Self & Informant Report Questionnaire		USA	Sasson et al (2013) [27]	ASD-P n=711 (50.5% Fa)	N-P n = 981 (49.9% Fa)	**ASD-Fa > N-Fa****<br>**ASD-Mo > N-Mo*****	**ASD-Fa vs. N-Fa 0.26**<br>**ASD-Mo vs. N-Mo 0.34**

	Rigid	USA	Hurley et al (2007) [26]	ASD-P = 86 (40Mo/46Fa) *BAP(+) n = 27* *BAP(-) n = 59*	N-P = 64 (32Mo/32Fa)	**BAP(+) > BAP(-), N-P*** **	**BAP(+) vs. BAP(-) 0.77 BAP(+) vs. N-P 0.73**
		USA	Sasson et al (2013) [27]	ASD-P n=711 (50.5% Fa)	N-P n = 981 (49.9% Fa)	**ASD-Fa > N-Fa*** ASD-Mo > N-Mo*** **	**ASD-Fa vs. N-Fa 0.35 ASD-Mo vs. N-Mo 0.29**
	Pragmatic Language	USA	Hurley et al (2007) [26]	ASD-P = 86 (40Mo/46Fa) *BAP(+) n = 27* *BAP(-) n = 59*	N-P = 64 (32Mo/32Fa)	**BAP(+) > BAP(-)** , N-P***	**BAP(+) vs. BAP(-) 0.94 BAP(+) vs. N-P 1.13**
		USA	Sasson et al (2013) [27]	ASD-P n=711 (50.5% Fa)	N-P n = 981 (49.9% Fa)	**ASD-Fa > N-Fa** ASD-Mo > N-Mo*** **	**ASD-Fa vs. N-Fa 0.28 ASD-Mo vs. N-Mo 0.44**
	Total score	USA	Hurley et al (2007) [26]	ASD-P = 86 (40Mo/46Fa) *BAP(+) n = 27* *BAP(-) n = 59*	N-P = 64 (32Mo/32Fa)	**BAP(+) > BAP(-), N-P*** **	**BAP(+) vs. BAP(-) 1.49 BAP(+) vs. N-P 1.43**
		USA	Sasson et al (2013) [27]	ASD-P n=711 (50.5% Fa)	N-P n = 981 (49.9% Fa)	**ASD-Fa > N-Fa*** ASD-Mo > N-Mo*** **	**ASD-Fa vs. N-Fa 0.37 ASD-Mo vs. N-Mo 0.45**
**Broader Phenotype Autism Symptom Scale (BPASS)** *Interview & Direct Behavioral Observation*	Social	USA	Bernier et al (2012) [28]	MPX-P n=39 SPX-P n=22	DD-P n = 20 TD-P n = 20	**MPX-P > SPX-P* > DD-P** > TD-P***	**MPX-P vs. DD-P 0.84 MPX-P vs. TD-P 0.77 MPX-P vs. SPX-P 0.75**
	Expressiveness	USA	Bernier et al (2012) [28]	MPX-P n=39 SPX-P n=22	DD-P n = 20 TD-P n = 20	**MPX-P > TD-P*** **	**MPX-P vs. TD-P 1.28**
	Conversation	USA	Bernier et al (2012) [28]	MPX-P n=39 SPX-P n=22	DD-P n = 20 TD-P n = 20	**n.s.**	
	Restricted Interests	USA	Bernier et al (2012) [28]	MPX-P n=39 SPX-P n=22	DD-P n = 20 TD-P n = 20	**MPX-P > TD-P***	**MPX-P vs. TD-P 0.93**
**Communication Checklist - Adult Version (CC-A)** *Informant Report*	Language Structure	UK / Ireland / USA / Canada / Australia	Whitehouse et al (2010) [29]	ASD-P n= 238 (115Mo/123Fa)	UA n= 187 (90M/97F)	**n.s.**	**ASD-P vs. UA 0.04**
	Pragmatic Skills	UK / Ireland / USA / Canada / Australia	Whitehouse et al (2010) [29]	ASD-P n= 238 (115Mo/123Fa)	UA n= 187 (90M/97F)	**n.s.**	**ASD-P vs. UA 0.18**

Questionnaire	Social Engagement	UK / Ireland / USA / Canada / Australia	Whitehouse et al (2010) [29]	ASD-P n= 238 (115Mo/123Fa)	UA n= 187 (90M/97F)	**ASD-P < UA***	**ASD-P vs. UA 0.43**
	Total Score (1 SD below mean)	UK / Ireland / USA / Canada / Australia	Whitehouse et al (2010) [29]	ASD-P n= 238 (115Mo/123Fa)	UA n= 187 (90M/97F)	**ASD-P < UA***	
**Family History Interview / Schedule (FHI/FHS)**	Social	USA	(FHS) Piven et al (1997a) [6]	MPX-P n= 48 (25Mo/23Fa)	DS-P n = 60 (30Mo/30Fa)	**MPX-P > DS-P**** **MPX-Fa > DS-Fa***** **MPX-Mo > DS-Mo***	
Interview		UK	Pickles et al (2013) [31] *Modified version*	ASD-P n = 193 (97Mo/96Fa)	SLI-P n = 103 (54Mo/49Fa) SLI+ASD-P n = 43 (23Mo/20Fa) DS-P n = 70 (35Mo/35Fa)	**ASD-P >SLI-P***	
	Communication	USA	(FHS) Piven et al (1997a) [6]	MPX-P n= 48 (25Mo/23Fa)	DS-P n = 60 (30Mo/30Fa)	**n.s.**	
		UK	Pickles et al (2013) [31] *Modified version*	ASD-P n = 193 (97Mo/96Fa)	SLI-P n = 103 (54Mo/49Fa) SLI+ASD-P n = 43 (23Mo/20Fa) DS-P n = 70 (35Mo/35Fa)	**n.s.**	
	Definite & Probable ELRCD	USA	Folstein et al (1999) [30]	ASD-P n = 166	DS-P n = 75	**ASD-P > DS-P****	
	Definite only ELRCD	USA	Folstein et al (1999) [30]	ASD-P n = 166	DS-P n = 75	**ASD-P > DS-P***	
	Stereotyped behavioours	USA	(FHS) Piven et al (1997a) [6]	MPX-P n= 48 (25Mo/23Fa)	DS-P n = 60 (30Mo/30Fa)	**MPX-P > DS-P***	
**The Modified Personality Assessment Schedule –**	Aloof	USA	Losh et al (2008) [34]	MPX n=48 (25Mo/23Fa) SPX n=78	DS-P n=60	**MPX-P > DS-P**** **SPX-P > DS-P**** **MPX-P > SPX-P***	

**Revised (MPAS-R)**<br><br>*Interview*		USA	Losh et al (2012) [35]	ASD-Mo n = 89 (All Mo)	FXS-Mo n = 49 (All Mo)<br>TD-Mo n = 23 (All Mo)	**n.s.**
		USA	(MPAS) Piven et al (1994) [32]	ASD-P n = 87 (45Mo/42Fa)	DS-P n = 38 (19Mo/19Fa)	**ASD-P > DS-P****
		USA	Piven et al (1997b) [33]	MPX-P n= 39	DS-P n = 58	**MPX-P > DS-P****
	Anxious	USA	Losh et al (2008) [34]	MPX-P n=48 (25Mo/23Fa)<br>SPX-P n=78	DS-P n=60	**MPX-P > DS-P****<br>**SPX-P > DS-P***<br>**MPX-P > SPX-P****
		USA	Piven et al (1997b) [33]	MPX-P n= 39	DS-P n = 58	**MPX-P > DS-P****<br>**MPX-Fa > DS-Fa****
	Hypersensitive	USA	Losh et al (2008) [34]	MPX-P n=48 (25Mo/23Fa)<br>SPX-P n=78	DS-P n=60	**MPX-P > DS-P*****<br>**SPX-P > DS-P***<br>**MPX-P > SPX-P***
		USA	Piven et al (1997b) [33]	MPX-P n= 39	DS-P n = 58	**MPX-P > DS-P****
	Overly conscientious	USA	Losh et al (2008) [34]	MPX n=48 (25Mo/23Fa)<br>SPX n=78	DS-P n=60	**MPX-P > DS-P***<br>**SPX-P > DS-P***
		USA	Losh et al (2012) [35]	ASD-Mo n = 89 (All Mo)	FXS-Mo n = 49 (All Mo)<br>TD-Mo n = 23 (All Mo)	**n.s.**
		USA	Piven et al (1997b) [33]	MPX-P n= 39	DS-P n = 58	**n.s.**
	Rigid	USA	Losh et al (2008) [34]	MPX-P n=48 (25Mo/23Fa)<br>SPX-P n=78	DS-P n=60	**MPX-P > DS-P*****<br>**SPX-P > DS-P***<br>**MPX-P > SPX-P****
		USA	Losh et al (2012) [35]	ASD-Mo n = 89 (All Mo)	FXS-Mo n = 49 (All Mo)<br>TD-Mo n = 23 (All Mo)	**ASD-P, FXS-P > TD-P***

		USA	Piven et al (1997b) [6]	MPX-P n= 39	DS-P n = 58	**MPX-P > DS-P****	
	Untactful	USA	Losh et al (2008) [34]	MPX-Pn=48 (25Mo/23Fa) SPX-P n=78	DS-P n=60	**MPX-P > DS-P*** **MPX-P > SPX-P***	
		USA	(MPAS) Piven et al (1994) [32]	ASD-P n = 87 (45Mo/42Fa)	DS-P n = 38 (19Mo/19Fa)	**ASD-P > DS-P***	
		USA	Piven et al (1997b) [33]	MPX-P n= 39	DS-P n = 58	**n.s.**	
	Undemonstrative	USA	(MPAS) Piven et al (1994) [32]	ASD-P n = 87 (45Mo/42Fa)	DS-P n = 38 (19Mo/19Fa)	**ASD-P > DS-P***	
		USA	Piven et al (1997b) [33]	MPX-P n= 39	DS-P n = 58	**n.s.**	
	Unresponsive	USA	Piven et al (1997b) [33]	MPX-P n= 39	DS-P n = 58	**n.s.**	
**Pragmatic Rating Scale (PRS)** *Interview & Direct Behavioral Observation*	Pragmatic language violations	USA	Losh et al (2008) [34]	MPX-P n=48 (25Mo/23Fa) SPX-P n=78	DS-P n=60	**SPX-P > DS-P****	
		USA	Losh et al (2012) [35]	ASD-Mo n = 89 (All Mo)	FXS-Mo n = 49 (All Mo) TD-Mo n = 23 (All Mo)	**ASD-Mo/FXS-Mo >TD-Mo***	
		USA	Piven et al (1997b) [33]	MPX-P n= 38	DS-P n = 58	**MPX-P > DS-P****	**MPX-P vs. DS-P 0.80**
	Speech errors	USA	Losh et al (2008) [34]	MPX-P n=48 (25Mo/23Fa) SPX-P n=78	DS-P n=60	**SPX-P > DS-P****	
		USA	Piven et al (1997b) [33]	MPX-P n= 38	DS-P n = 58	**MPX-P > DS-P****	**MPX-P vs. DS-P 0.93**
	Total score (blind ratings)	USA	Landa et al (1992) [36]	ASD-P n = 21	TD/DS n = 19	**ASD-P > TD/DS-P***	**ASD-P vs. TD/DS-P 0.71**
	Total score (blind &	USA	Landa et al	ASD-P n = 43	TD/DS n = 21	**ASD-P > TD/DS-P*****	**ASD-P vs. TD/DS-P 0.87**

	unblind ratings combined)		(1992) [36]		TD n = 11<br>DS n = 10		
**Pragmatic Rating Scale - Modified (PRS-M)**	Emotional expressiveness and awareness of the other	USA	Ruser et al (2007) [37]	ASD-P n= 47 (49% Fa)	SLI-P n= 47 (45% Fa)<br>DS-P n = 21 (48% Fa)	**ASD-P > DS-P***	**ASD-P vs. SLI-P 0.25**<br>**ASD-P vs. DS-P 0.58**
Interview & Direct Behavioral Observation	Communicative performance	USA	Ruser et al (2007) [37]	ASD-P n= 47 (49% Fa)	SLI-P n= 47 (45% Fa)<br>DS-P n = 21 (48% Fa)	**n.s.**	**ASD-P vs. SLI-P 0.06**<br>**ASD-P vs. DS-P 0.40**
	Over-talkativeness	USA	Ruser et al (2007) [37]	ASD-P n= 47 (49% Fa)	SLI-P n= 47 (45% Fa)<br>DS-P n = 21 (48% Fa)	**ASD-P > DS-P***	**ASD-P vs. SLI-P 0**<br>**ASD-P vs. DS-P 0.53**
	Language	USA	Ruser et al (2007) [37]	ASD-P n= 47 (49% Fa)	SLI-P n= 47 (45% Fa)<br>DS-P n = 21 (48% Fa)	**ASD-P > DS-P****	**ASD-P vs. SLI-P 0.14**<br>**ASD-P vs. DS-P 0.92**
	Total score	USA	Ruser et al (2007) [37]	ASD-P n= 47 (49% Fa)	SLI-P n= 47 (45% Fa)<br>DS-P n = 21 (48% Fa)	**ASD-P > DS-P****	**ASD-P vs. SLI-P 0.09**<br>**ASD-P vs. DS-P 1.14**
**Social Responsiveness Scale (SRS)**<br>*Self & Informant Report Questionnaire*		Belgium / Netherlands	De la Marche et al (2012) [38]	ASD-P n = 275 (143Mo/132Fa)<br>MPX-P n = 93 (48Mo/45Fa)<br>SPX-P n = 129 (68Mo/61Fa)	UA n = 595 (295F/300M)	**ASD-Fa > UA-M****<br>**MPX-Fa > UA-M***	**ASD-Fa vs. UA-M 0.30**<br>**ASD-Mo vs. UA-F 0.28**<br>**MPX-Fa vs. UA-M 0.44**<br>**SPX-Fa vs. UA-M 0.19**<br>**MPX-Fa vs. SPX-Fa 0.23**
		USA	Schwichtenberg et al (2010) [39]	MPX-P n = 21 (10Mo/11Fa)<br>SPX-P n = 239 (115Mo/124Fa)	TD-P n = 163 (81Mo/82Fa)	**MPX-Fa > TD-Fa***<br>**SPX-Fa > TD-Fa***	**MPX-Fa vs. TD-Fa 0.90**<br>**SPX-Fa vs. TD-Fa 0.35**<br>**MPX-Fa vs. SPX-Fa 0.38**<br>**MPX-Mo vs. TD-Mo 0.27**<br>**SPX-Mo vs. TD-Mo 0.02**<br>**MPX-Mo vs. SPX-Mo 0.18**

**Other measures of Personality and Friendships**	**The Friendship Interview**	Quality of friendships (higher scores indicate fewer friendships)	USA	Losh et al (2008) [34]	MPX-P n=48 (25Mo/23Fa) SPX-P n=78	DS-P n=60	**SPX-P > DS-P**** **MPX-P > SPX-P***	
			USA	Piven et al (1997b) [33]	MPX-P n= 38	DS-P n = 58	**MPX-P > DS-P*****	**MPX-P vs. DS-P 1.14**
	**The NEO Personality Inventory (NEO-PI)**	Neuroticism	USA	Losh et al (2008) [34]	MPX-P n=48 (25Mo/23Fa) SPX-P n=78	DS-P n=60	**SPX-P > DS-P***** **MPX-P > SPX-P***	
			USA	Piven et al (1997b) [33]	MPX-P n= 38	DS-P n = 58	**MPX-P > DS-P*****	**MPX-P vs. DS-P 0.79**
**Reciprocal Social Interaction** *Alexithymia*	**Toronto Alexithymia Scale (TAS-20)**	Difficulty Identifying Feelings (DIF)	Canada	Szatmari et al (2008) [9]	ASD-P n = 439 (237Mo/202Fa)	PW-P n = 45 (28Mo/17Fa)	**n.s.**	
			UK	Berthoz et al (2013) [40]	ASD-P n = 87 (28%Fa)	UA n = 47 (62%M)	**n.s.**	**ASD-P vs. UA 0.25**
		Difficulty Describing Feelings (DDF)	Canada	Szatmari et al (2008) [9]	ASD-P n = 439 (237Mo/202Fa)	PW-P n = 45 (28Mo/17Fa)	**n.s.**	
			UK	Berthoz et al (2013) [40]	ASD-P n = 87 (28%Fa)	UA n = 47 (62%M)	**n.s.**	**ASD-P vs. UA 0.14**
		Externally-Oriented Thinking (EOT)	Canada	Szatmari et al (2008) [9]	ASD-P n = 439 (237Mo/202Fa)	PW-P n = 45 (28Mo/17Fa)	**n.s.**	
			UK	Berthoz et al (2013) [40]	ASD-P n = 87 (28%Fa)	UA n = 47 (62%M)	**n.s.**	**ASD-P vs. UA 0.16**
		Total score	Canada	Szatmari et al (2008) [9]	ASD-P n = 439 (237Mo/202Fa)	PW-P n = 45 (28Mo/17Fa)	**ASD-P > PW-P***	
			UK	Berthoz et al	ASD-P n = 87	UA n = 47	**n.s.**	**ASD-P vs. UA 0.23**

				(2013) [40]	(28%Fa)	(62%M)		
	**Bermond-Vorst Alexithymia Questionnire-B (BVAQ-B)**	Total score	UK	Berthoz et al (2013) [40]	ASD-P n = 87 (28%Fa)	UA n = 47 (62%M)	**n.s.**	**ASD-P vs. UA 0.15**
		Cognitive score	UK	Berthoz et al (2013) [40]	ASD-P n = 87 (28%Fa)	UA n = 47 (62%M)	**n.s.**	**ASD-P vs. UA 0.19**
		Affective score	UK	Berthoz et al (2013) [40]	ASD-P n = 87 (28%Fa)	UA n = 47 (62%M)	**n.s.**	**ASD-P vs. UA 0.02**
Anhedonia	**Revised Social Anhedonia Scale (SAS)**	Total score	UK	Berthoz et al (2013) [40]	ASD-P n = 87 (28%Fa)	UA n = 47 (62%M)	**n.s.**	**ASD-P vs. UA 0.25**
	**Physical Anhedonia Scale (PAS)**	Total score	UK	Berthoz et al (2013) [40]	ASD-P n = 87 (28%Fa)	UA n = 47 (62%M)	**ASD-P > UA****	**ASD-P vs. UA 0.33**
**Social and Narrative Language**	**Grice's Conversational Maxims Task**	No. of errors	Italy	Di Michele et al (2007) [8]	ASD n = 46	DS n=14<br>TD n=12	**ASD-P > TD-P*****<br>**ASD-P > DS-P***	
	**Spontaneous Narrative Discourse**	Story length (mean o. of clauses)	USA	Landa et al (1991) [41]	ASD-P n = 41	TD/DS-P n = 23 total<br>TD-P n = 10<br>DS-P n = 13	**n.s.**	**ASD-P vs. TD/DS-P 0.35**
		Stories w/ complete episodes	USA	Landa et al (1991) [41]	ASD-P n = 41	TD/DS-P n = 23 total<br>TD-P n = 10<br>DS-P n = 13	**ASD-P < TD/DS-P****	**ASD-P vs. TD/DS-P 0.71**
		Stories w/ multiple episodes	USA	Landa et al (1991) [41]	ASD-P n = 41	TD/DS-P n = 23 total<br>TD-P n = 10<br>DS-P n = 13	**ASD-P < TD/DS-P****	
		Stories w/ incomplete episodes	USA	Landa et al (1991) [41]	ASD-P n = 41	TD/DS-P n = 23 total<br>TD-P n = 10	**ASD-P > TD/DS-P***	**ASD-P vs. TD/DS-P 0.50**

						DS-P n = 13		
		Mean overall quality	USA	Landa et al (1991) [41]	ASD-P n = 41	TD/DS-P n = 23 total<br>TD-P n = 10<br>DS-P n = 13	**ASD-P < TD/DS-P****	**ASD-P vs. TD/DS-P 0.73**
**Repetitive, restrictive behaviors and interests**	**Real-life Skills and Preferences**	Social items	UK	Briskman et al (2001) [42]	ASD-P n = 42 (21Mo/21Fa)	DLX-P n = 27 (14Mo/13Fa)<br>TD-P n = 28 (14Mo/14Fa)	**ASD-P > DLX-P > TD-P***<br>**ASD-Fa > DLX-Fa****<br>**ASD-Fa > TD-Fa****<br>**ASD-Mo > DLX-Mo****<br>**ASD-Mo > TD-Mo****	**ASD-P vs. DLX-P 0.92**<br>**ASD-P vs. TD-P 0.91**<br>**ASD-Fa vs. DLX-Fa 1.03**<br>**ASD-Fa vs. TD-Fa 1.11**<br>**ASD-Mo vs. DLX-Mo 0.89**<br>**ASD-Mo vs. TD-Mo 0.77**
(Everyday Preferences and abilities)		Non-social items	UK	Briskman et al (2001) [42]	ASD-P n = 42 (21Mo/21Fa)	DLX-P n = 27 (14Mo/13Fa)<br>TD-P n = 28 (14Mo/14Fa)	**ASD-P > TD-P****<br>**ASD-Fa > TD-Fa***	**ASD-P vs. DLX-P 0.44**<br>**ASD-P vs. TD-P 0.76**<br>**ASD-Fa vs. DLX-Fa 0.37**<br>**ASD-Fa vs. TD-Fa 0.9**<br>**ASD-Mo vs. DLX-Mo 0.54**<br>**ASD-Mo vs. TD-Mo 0.64**

Note: ASD, Autism Spectrum Disorder; BAP, Broad Autism Phenotype; BAP(+), BAP present; BAP (-), BAP absent; P, Parent; Mo, Mother; Fa, Father; M, Male; F, Female; MPX, Multiple incidence autism families; SPX, Single incidence autism families; DD, Developmental delay without autism; DLX, Dyslexia; DS, Down Syndrome; FXS, Fragile X Syndrome; N, Normative sample; PWD, Prader Willie; SLI, Specific Language Impairment; TD, typically developing; UA, Unaffected adult

*p<0.05; **p<0.01; ***p<0.001

Supplementary Table 2. Cognitive Endophenotype Matrix – Review of studies of parents of autistic probands.

Domain	Method / Measure	Factors / Subscales	Country	Study	ASD Parent Group Characteristics	Control Group Characteristics	Key Findings in relation to Proband Diagnosis	
							P value	Effect Size (*d*)
**General Intellectual Functioning**	***Weschler Scales*** Verbal IQ (VIQ)	Total Score or Estimate	Australia	Bishop et al (2004a) [20]	ASD-P n = 121 (69Mo/52Fa)	N-P n = 89 (52Mo/37Fa)	**n.s.**	**ASD-Mo vs. N-Mo 0.20**<br>**ASD-Fa vs. N-Fa 0.21**
			Australia	Bishop et al (2004b) [53]	ASD-P = 142 (77Mo/65Fa)	N-P n = 96 (57Mo/39Fa)	**n.s.**	**ASD-Mo vs. N-Mo 0.19**<br>**ASD-Fa vs. N-Fa 0.07**
			Netherlands	de Jonge et al (2006) [65]	MPX-P n = 51 (26Mo/25Fa)	DS-P n = 54 (28Fa/26Mo)	**n.s.**	**MPX-P vs. DS-P 0.11**
			Netherlands	de Jonge et al (2007) [67]	MPX-P n = 51 (26Mo/25Fa)	DS-P n = 52 (25Fa/27Mo)	**n.s.**	**MPX-P vs. DS-P 0.17**
			Netherlands	de Jonge et al (2009) [66]	MPX-P n = 51 (26Mo/25Fa)	DS-P n = 57 (28Mo/29Fa)	**n.s.**	**MPX-P vs. DS-P 0.09**
			USA	Folstein et al (1999) [30]	ASD-P n = 166	DS-P n = 75	**n.s.**	**ASD-P vs. DS-P 0.19**
			UK	Fombonne et al (1997) [44]	ASD-P n = 160 (86Mo/74Fa)	DS-P n = 42 (23Mo/19Fa)	**ASD-P > DS-P****	**ASD-P vs. DS-P 0.51**
			UK	Happé et al (2001) [64]	ASD-P n = 43 (21Mo/22Fa)	DLX-P n = 30 (15Mo/15Fa) TD-P n = 20 (10Mo/10Fa)	**n.s.**	**ASD-Mo vs. DLX-Mo 0.05**<br>**ASD-Mo vs. TD-Mo 0.17**<br>**ASD-Fa vs. DLX-Fa 0.26**<br>**ASD-Fa vs. TD-Fa 0.19**
			USA	Lindgren et al (2009) [47]	ALN-P n = 39 (20Mo/19Fa) ALI-P n = 62 (31Mo/31Fa)	SLI-P n = 70 (35Mo/35Fa)	**n.s.**	**ALN-Fa vs. ALI-Fa 0.95**<br>**ALN-Fa vs. SLI-Fa 2.10**<br>**ALI-Fa vs. SLI-Fa 0.95**<br>**ALN-Mo vs. ALI-Mo 0.80**<br>**ALN-Mo vs. SLI-Mo 2.10**<br>**ALI-Mo vs. SLI-Mo 1.28**
			USA	Piven & Palmer (1997) [46]	MPX-P n= 48 (25Mo/23Fa)	DS-P n = 60 (30Mo/30Fa)	**n.s.**	**MPX-P vs. DS-P 0.16**
			USA	Ruser et al (2007) [37]	ASD-P n= 47 (49% Fa)	SLI-P n= 47 (45% Fa) DS-P n = 21	**n.s.**	**ASD-P vs. SLI-P 0.09**<br>**ASD-P vs. DS-P 0.26**

				(48% Fa)		
	USA	Schmidt et al (2008) [10]	ASD-P n = 22 (14Mo/8Fa)	UA n = 22 (14F/8M)	**n.s.**	**ASD-P vs. UA 0.07**
	Australia	Wong et al (2006) [62]	ASD-P n = 145 (80Mo/65Fa)	TD-P n = 96 (57Mo/39Fa)	**n.s.**	
Digit Span	UK	Fombonne et al (1997) [44]	ASD-P n = 160 (86Mo/74Fa)	DS-P n = 42 (23Mo/19Fa)	**ASD-P > DS-P****	**ASD-P vs. DS-P 0.48**
	UK	Whitehouse et al (2007) [23] (modified version to assess short-term memory)	ASD-P n = 30 (20Mo/10Fa)	SLI-P n= 30 (22Mo/8Fa) TD-P n = 30 (23Mo/7Fa)	**ASD-P > SLI-P****	**ASD-P vs. SLI-P 0.67** **ASD-P vs. TD-P 0.14**
	Canada	Szatmari et al (1993) [76]	ASD-P n = 97 (51Mo/46Fa)	DS/LBW-P n = 54 (30Mo/24Fa)	**n.s.**	**ASD-Fa vs. DS/LBW-P 0.40** **ASD-M0 vs. DS/LBW-P 0.04**
Arithmetic	UK	Fombonne et al (1997) [44]	ASD-P n = 160 (86Mo/74Fa)	DS-P n = 42 (23Mo/19Fa)	**n.s.**	**ASD-P vs. DS-P 0.25**
Vocabulary	UK	Fombonne et al (1997) [44]	ASD-P n = 160 (86Mo/74Fa)	DS-P n = 42 (23Mo/19Fa)	**ASD-P > DS-P*****	**ASD-P vs. DS-P 0.58**
	USA	Schmidt et al (2008) [10]	ASD-P n = 22 (14Mo/8Fa)	UA n = 22 (14F/8M)	**n.s.**	**ASD-P vs. UA 0.24**
	USA	Smalley & Asarnow (1990) [45]	ASD-P n = 15	TD-P n = 12	**ASD-P < TD-P***	**ASD-P vs. TD-P 0.96**
	Canada	Szatmari et al (1993) [76]	ASD-P n = 97 (51Mo/46Fa)	DS/LBW-P n = 54 (30Mo/24Fa)	**n.s.**	**ASD-Fa vs. DS/LBW-P 0.40** **ASD-M0 vs. DS/LBW-P 0.04**
Comprehension	UK	Fombonne et al (1997) [44]	ASD-P n = 160 (86Mo/74Fa)	DS-P n = 42 (23Mo/19Fa)	**ASD-P > DS-P***	**ASD-P vs. DS-P 0.35**
	USA	Smalley & Asarnow (1990) [45]	ASD-P n = 15	TD-P n = 12	**n.s.**	**ASD-P vs. TD-P 0.74**

		Canada	Szatmari et al (1993) [76]	ASD-P n = 97 (51Mo/46Fa)	DS/LBW-P n = 54 (30Mo/24Fa)	**n.s.**	**ASD-Fa vs. DS/LBW-P 0.36**<br>**ASD-M0 vs. DS/LBW-P 0.31**
		Iran	Tajmirriyahi et al (2013) [58]	ASD-P n = 48 (38Mo/10Fa)	DS-P n = 31 (25Mo/6Fa)<br>TD-P n = 30 (23Mo/7Fa)	**n.s.**	**ASD-P vs. DS-P 0.42**<br>**ASD-P vs. TD-P 0.39**
	Similarities	UK	Fombonne et al (1997) [44]	ASD-P n = 160 (86Mo/74Fa)	DS-P n = 42 (23Mo/19Fa)	**ASD-P > DS-P***	**ASD-P vs. DS-P 0.35**
		USA	Schmidt et al (2008) [10]	ASD-P n = 22 (14Mo/8Fa)	UA n = 22 (14F/8M)	**n.s.**	**ASD-P vs. UA 0.13**
Performance IQ (PIQ)	Total Score or Estimate	Australia	Bishop et al (2004a) [20]	ASD-P n = 121 (69Mo/52Fa)	N-P n = 89 (52Mo/37Fa)	**n.s.**	**ASD-Mo vs. N-Mo 0.10**<br>**ASD-Fa vs. N-Fa 0.17**
		Australia	Bishop et al (2004b) [53]	ASD-P = 142 (77Mo/65Fa)	N-P n = 96 (57Mo/39Fa)	**n.s.**	**ASD-Mo vs. N-Mo 0.03**<br>**ASD-Fa vs. N-Fa 0.38**
		Netherlands	de Jonge et al (2006) [65]	MPX-P n=51 (26Mo/25Fa)	DS-P n = 54 (28Fa/26Mo)	**n.s.**	**MPX-P vs. DS-P 0.03**
		Netherlands	de Jonge et al (2007) [67]	MPX-P n = 51 (26Mo/25Fa)	DS-P n = 52 (25Fa/27Mo)	**n.s.**	**MPX-P vs. DS-P 0.11**
		Netherlands	de Jonge et al (2009) [66]	MPX-P n = 51 (26Mo/25Fa)	DS-P n = 57 (28Mo/29Fa)	**n.s.**	**MPX-P vs. DS-P 0.05**
		USA	Folstein et al (1999) [30]	ASD-P n = 166	DS-P n = 75	**ASD-P < DS-P****<br>**ASD-Fa < DS-Fa***	**ASD-P vs. DS-P 0.35**
		UK	Fombonne et al (1997) [44]	ASD-P n = 160 (86Mo/74Fa)	DS-P n = 42 (23Mo/19Fa)	**n.s.**	**ASD-P vs. DS-P 0.13**
		UK	Happé et al (2001) [64]	ASD-P n = 43 (21Mo/22Fa)	DLX-P n = 30 (15Mo/15Fa)<br>TD-P n = 20 (10Mo/10Fa)	**n.s.**	**ASD-Mo vs. DLX-Mo 0.09**<br>**ASD-Mo vs. TD-Mo 0.26**<br>**ASD-Fa vs. DLX-Fa 0.14**<br>**ASD-Fa vs. TD-Fa 0**

	USA	Lindgren et al (2009) [47]	ALN-P n = 39 (20Mo/19Fa)<br>ALI-P n = 62 (31Mo/31Fa)	SLI-P n = 70 (35Mo/35Fa)	**ALN-Fa > ALI-Fa > SLI-Fa***	**ALN-Fa vs. ALI-Fa 0.44**<br>**ALN-Fa vs. SLI-Fa 1.16**<br>**ALI-Fa vs. SLI-Fa 0.72**<br>**ALN-Mo vs. ALI-Mo 0.37**<br>**ALN-Mo vs. SLI-Mo 0.77**<br>**ALI-Mo vs. SLI-Mo 0.40**
	USA	Piven & Palmer (1997) [46]	MPX-P n= 48 (25Mo/23Fa)	DS-P n = 60 (30Mo/30Fa)	**MPX-P < DS-P***	**MPX-P vs. DS-P 0.74**
	USA	Ruser et al (2007) [37]	ASD-P n= 47 (49% Fa)	SLI-P n= 47 (45% Fa)<br>DS-P n = 21 (48% Fa)	**n.s.**	**ASD-P vs. SLI-P 0.14**<br>**ASD-P vs. DS-P 0.48**
	USA	Schmidt et al (2008) [10]	ASD-P n = 22 (14Mo/8Fa)	UA n = 22 (14F/8M)	**ASD-P< UA***	**ASD-P vs. UA 0.62**
	Australia	Wong et al (2006) [62]	ASD n = 145 (80Mo/65Fa)	TD-P n = 96 (57Mo/39Fa)	**n.s.**	
Picture Completion	USA	Folstein et al (1999) [30]	ASD-P n = 166	DS-P n = 75	**ASD-P < DS-P****	**ASD-P vs. DS-P 0.42**
	UK	Fombonne et al (1997) [44]	ASD-P n = 160 (86Mo/74Fa)	DS-P n = 42 (23Mo/19Fa)	**n.s.**	**ASD-P vs. DS-P 0.07**
	USA	Piven & Palmer (1997) [46]	MPX-P n= 48 (25Mo/23Fa)	DS-P n = 60 (30Mo/30Fa)	**MPX-P < DS-P***	**MPX-P vs. DS-P 0.65**
	Iran	Tajmirriyahi et al (2013) [58]	ASD-P n = 48 (38Mo/10Fa)	DS-P n = 31 (25Mo/6Fa)<br>TD-P n = 30 (23Mo/7Fa)	**n.s.**	**ASD-P vs. DS-P 0.12**<br>**ASD-P vs. TD-P 0.46**
Picture Arrangement	USA	Folstein et al (1999) [30]	ASD-P n = 166	DS-P n = 75	**ASD-P < DS-P***	**ASD-P vs. DS-P 0.26**
	UK	Fombonne et al (1997) [44]	ASD-P n = 160 (86Mo/74Fa)	DS-P n = 42 (23Mo/19Fa)	**n.s.**	**ASD-P vs. DS-P 0.03**

	Block Design	UK	Fombonne et al (1997) [44]	ASD-P n = 160 (86Mo/74Fa)	DS-P n = 42 (23Mo/19Fa)	**n.s.**	**ASD-P vs. DS-P 0.21**
		USA	Piven & Palmer (1997) [46]	MPX-P n= 48 (25Mo/23Fa)	DS-P n = 60 (30Mo/30Fa)	**n.s.**	**MPX-P vs. DS-P 0.34**
		USA	Schmidt et al (2008) [10]	ASD-P n = 22 (14Mo/8Fa)	UA n = 22 (14F/8M)	**n.s.**	**ASD-P vs. UA 0.43**
		USA	Smalley & Asarnow (1990) [45]	ASD-P n = 15	TD-P n = 12	**n.s.**	**ASD-P vs. TD-P 0.36**
		Canada	Szatmari et al (1993) [76]	ASD-P n = 97 (51Mo/46Fa)	DS/LBW-P n = 54 (30Mo/24Fa)	**n.s.**	**ASD-Fa vs. DS/LBW-P 0.38**<br>**ASD-M0 vs. DS/LBW-P 0.04**
	Object Assembly	UK	Fombonne et al (1997) [44]	ASD-P n = 160 (86Mo/74Fa)	DS-P n = 42 (23Mo/19Fa)	**n.s.**	**ASD-P vs. DS-P 0.12**
		USA	Piven & Palmer (1997) [46]	MPX-P n= 48 (25Mo/23Fa)	DS-P n = 60 (30Mo/30Fa)	**MPX-P < DS-P***	**MPX-P vs. DS-P 0.62**
	Matrix Reasoning	USA	Schmidt et al (2008) [10]	ASD-P n = 22 (14Mo/8Fa)	UA n = 22 (14F/8M)	**ASD-P < UA***	**ASD-P vs. UA 0.67**
	Digit Symbol	Canada	Szatmari et al (1993) [76]	ASD-P n = 97 (51Mo/46Fa)	DS/LBW-P n = 54 (30Mo/24Fa)	**n.s.**	**ASD-Fa vs. DS/LBW-P 0.19**<br>**ASD-M0 vs. DS/LBW-P 0.17**
Full Scale IQ (FSIQ)	Total Score or Estimate	USA	Adolphs et al (2008) [59]	AD-P n = 42 total<br>BAP(+) Aloof n = 15 (3Mo/12Fa)<br>BAP(-) n = 27 (20Mo/7Fa)	TD-P n = 20 (8Mo/12Fa)	**n.s.**	**BAP (+) Aloof vs. TD-P 0.47**<br>**BAP(-) vs. TD-P 0.37**
		Netherlands	de Jonge et al (2006) [65]	MPX-P n=51 (26Mo/25Fa)	DS-P n = 54 (28Fa/26Mo)	**n.s.**	**MPX-P vs. DS-P 0.09**
		Netherlands	de Jonge et al (2007) [67]	MPX-P n = 51 (26Mo/25Fa)	DS-P n = 52 (25Fa/27Mo)	**n.s.**	**MPX-P vs. DS-P 0.10**
		Netherlands	de Jonge et al (2009) [66]	MPX-P n = 51 (26Mo/25Fa)	DS-P n = 57 (28Mo/29Fa)	**n.s.**	**MPX-P vs. DS-P 0.06**

USA	Folstein et al (1999) [30]	ASD-P n = 166	DS-P n = 75	**ASD-P < DS-P**<br>ASD-Fa < DS-Fa***	**ASD-P vs. DS-P 0.30**
UK	Fombonne et al (1997) [44]	ASD-P n = 160 (86Mo/74Fa)	DS-P n = 42 (23Mo/19Fa)	**n.s.**	**ASD-P vs. DS-P 0.25**
Turkey	Gocken et al (2009) [57]	ASD-P n = 76 (38Mo/38Fa)	N-P n = 41 (21Mo/20Fa)	**n.s.**	**ASD-P vs. N-P 0.09**
UK	Happé et al (2001) [64]	ASD-P n = 43 (21Mo/22)	DLX-P n = 30 (15Mo/15Fa)<br>TD-P n = 20 (10Mo/10Fa)	**n.s.**	**ASD-Mo vs. DLX-Mo 0.05**<br>**ASD-Mo vs. TD-Mo 0.21**<br>**ASD-Fa vs. DLX-Fa 0.28**<br>**ASD-Fa vs. TD-Fa 0.11**
USA	Lindgren et al (2009) [47]	ALN-P n = 39 (20Mo/19Fa)<br>ALI-P n = 62 (31Mo/31Fa)	SLI-P n = 70 (35Mo/35Fa)	**n.s.**	**ALN-Fa vs. ALI-Fa 0.81**<br>**ALN-Fa vs. SLI-Fa 1.88**<br>**ALI-Fa vs. SLI-Fa 0.97**<br>**ALN-Mo vs. ALI-Mo 0.66**<br>**ALN-Mo vs. SLI-Mo 1.61**<br>**ALI-Mo vs. SLI-Mo 0.94**
USA	Losh & Piven (2007) [48]	ASD-P n = 48 (25Mo/23Fa)<br>BAP(+) A*loof* n = 13<br>BAP(+) R*igid* n = 11<br>BAP(-) n = 24	TD/DS-P n = 22<br>TD-P n = 16<br>DS-P n = 6	**ASD-P < TD/DS-P****	**ASD-P vs. TD/DS-P 0.83**
USA	Losh et al (2008) [34]	MPX n=48 (25Mo/23Fa)<br>SPX n=78	DS-P n=60	**MPX-P < DS-P***<br>**SPX-P < DS-P***	
USA	Losh et al (2009) [56]	ASD-P n= 83<br>BAP(+) *Social* n = 22<br>BAP(+) *Rigid/Perfectionistic* n = 34<br>BAP(+) *Social & Rigid* n = 13 (5Mo/8Fa)<br>BAP(-) n = 40	TD-P n = 32 (19Mo/13Fa)	**n.s.**	**ASD-P vs.TD-P 0.33**
USA	Piven et al (1991) [68]	ASD-P n = 81 (42Mo/39Fa)	DS-P n = 34 (18Mo/16Fa)	**n.s**	**ASD-P vs. DS-P 0.21**
USA	Schmidt et al	ASD-P n = 22	UA n = 22	**n.s.**	**ASD-P vs. UA 0.41**

				(2008) [10]	(14Mo/8Fa)	(14F/8M)		
	Nonvebal Reasoning	Block Design & Matrix Reasoning	UK	Whitehouse et al (2007) [23]	ASD-P n = 30 (20Mo/10Fa)	SLI-P n= 30 (22Mo/8Fa) TD-P n = 30 (23Mo/7Fa)	**n.s.**	**ASD-Fa vs. SLI-Fa 0.30** **ASD-Mo vs. SLI-Mo 0.43** **ASD-Fa vs. TD-Fa 0.01** **ASD-Mo vs. TD-Mo 0.53**
	Raven's Progressive Matrices Nonvebal IQ (NVIQ)	Total Score	Germany / Austria / Switzerland	Bölte & Poustka (2003) [49]	ASD SPX-P n = 54 (26Mo/ 28Fa) ASD MPX-P n = 28 (16Mo/12Fa)	Sch SPX-P n = 31 (18Mo/ 13Fa) Sch MPX-P n = 4 (2Mo/2Fa) UA n = 22 (11F/11M)	**n.s.**	**ASD MPX-P vs. Sch MPX-P 0.29** **ASD MPX vs. Sch SPX-P 0.07** **ASD MPX-P vs. UA 0.08** **ASD SPX-P vs. Sch MPX-P 0.28** **ASD SPX-P vs. Sch SPX-P 0.05** **ASD SPX-P vs. UA 0.07**
			Germany	Bölte & Poustka (2006) [50]	ASD n=62 (33Mo/29Fa)	EOS-P n = 36 (20Mo/16Fa) MR-P n = 30 (16Mo/14Fa)	**n.s.**	**ASD-P vs. EOS-P 0.54** **ASD-P vs. MR-P 0.45**
			Germany	Bölte et al (2007) [51]	ASD SPX-P n = 87 (48Mo/39Fa) ASD MPX-P n = 38 (21Mo/17Fa)	OCD-P n = 37 (19Mo/18Fa) EOS-P n = 34 (20Mo/14Fa) MR-P n = 27 (15Mo/12Fa)	**n.s.**	**MPX-P vs. OCD-P 0.13** **MPX-P vs. EOS-P 0.52** **MPX-P vs. MR-P 0.56** **SPX-P vs. OCD-p 0.06** **SPX-P vs. EOS-P 0.48** **SPX-P vs. MR-P 0.57**
			UK	Sucksmith et al (2013) [51]	ASD-P n = 310 (272Mo/38Fa)	UA n = 187 (93M/94F)	**n.s.**	**ASD-P vs. UA 0.17**
**Structural Language Abilities** ***Receptive Language***	Peabody Picture Vocabulary Test (PPVT-III)	Receptive language	USA	Lindgren et al (2009) [47]	ALN-P n = 39 (20Mo/19Fa) ALI-P n = 62 (31Mo/31Fa)	SLI-P n = 70 (35Mo/35Fa)	**ALN-Mo > ALI-Mo > SLI-Mo*** **	**ALN-Fa vs. ALI-Fa 0.77** **ALN-Fa vs. SLI-Fa 1.58** **ALI-Fa vs. SLI-Fa 0.74** **ALN-Mo vs. ALI-Mo 0.42** **ALN-Mo vs. SLI-Mo 1.33** **ALI-Mo vs. SLI-Mo 0.87**
			USA	Schmidt et al (2008) [10]	ASD-P n = 22 (14Mo/8Fa)	UA n = 22 (14F/8M)	**n.s.**	**ASD-P vs. UA 0.33**
	Test for Reception of Grammar-2	Receptive Grammar	UK	Whitehouse et al (2007) [23]	ASD-P n = 30 (20Mo/10Fa)	SLI-P n= 30 (22Mo/8Fa) TD-P n = 30	**n.s.**	

	(TROG-2)					(23Mo/7Fa)		
Expressive Language	Expressive Vocabulary Test (EVT)	Expressive language	USA	Schmidt et al (2008) [10]	ASD-P n = 22 (14Mo/8Fa)	UA n = 22 (14F/8M)	**n.s.**	**ASD-P vs. UA 0.10**
	Verbal Fluency Subtest - Delis Kaplan Executive Function System (DK-EFS)	Letter fluency	USA	Schmidt et al (2008) [10]	ASD-P n = 22 (14Mo/8Fa)	UA n = 22 (14F/8M)	**n.s.**	**ASD-P vs. UA 0.39**
		Category fluency	USA	Schmidt et al (2008) [10]	ASD-P n = 22 (14Mo/8Fa)	UA n = 22 (14F/8M)	**n.s.**	**ASD-P vs. UA 0.35**
		Category switching	USA	Schmidt et al (2008) [10]	ASD-P n = 22 (14Mo/8Fa)	UA n = 22 (14F/8M)	**n.s.**	**ASD-P vs. UA 0.16**
		Switching Accuracy	USA	Schmidt et al (2008) [10]	ASD-P n = 22 (14Mo/8Fa)	UA n = 22 (14F/8M)	**n.s.**	**ASD-P vs. UA 0.22**
Figurative Language	Figurative Language Subtest - Test of Language Competence-Expanded Edition (TOLC-E)	Figurative Language	USA	Schmidt et al (2008) [10]	ASD-P n = 22 (14Mo/8Fa)	UA n = 22 (14F/8M)	**n.s.**	**ASD-P vs. UA 0.28**
Phonological awareness	Comprehensive Test of Phonological Processing (CTOPP)	Phonological Awareness	USA	Lindgren et al (2009) [47]	ALN-P n = 39 (20Mo/19Fa) ALI-P n = 62 (31Mo/31Fa)	SLI-P n = 70 (35Mo/35Fa)	**ALN-Mo > ALI-Mo > SLI-Mo*****	**ALN-Fa vs. ALI-Fa 0.42<br>ALN-Fa vs. SLI-Fa 0.86<br>ALI-Fa vs. SLI-Fa 0.38<br>ALN-Mo vs. ALI-Mo 0.38<br>ALN-Mo vs. SLI-Mo 0.78<br>ALI-Mo vs. SLI-Mo 0.48**
		Phonological memory	USA	Lindgren et al (2009) [47]	ALN-P n = 39 (20Mo/19Fa) ALI-P n = 62 (31Mo/31Fa)	SLI-P n = 70 (35Mo/35Fa)	**n.s.**	**ALN-Fa vs. ALI-Fa 0.08<br>ALN-Fa vs. SLI-Fa 0.96<br>ALI-Fa vs. SLI-Fa 0.86<br>ALN-Mo vs. ALI-Mo 0.74<br>ALN-Mo vs. SLI-Mo 1.42**

							**ALI-Mo vs. SLI-Mo 0.70**
	Rapid naming	USA	Lindgren et al (2009) [47]	ALN-P n = 39 (20Mo/19Fa)<br>ALI-P n = 62 (31Mo/31Fa)	SLI-P n = 70 (35Mo/35Fa)	**n.s.**	**ALN-Fa vs. ALI-Fa 0.02**<br>**ALN-Fa vs. SLI-Fa 0.34**<br>**ALI-Fa vs. SLI-Fa 0.36**<br>**ALN-Mo vs. ALI-Mo 0.09**<br>**ALN-Mo vs. SLI-Mo 0.47**<br>**ALI-Mo vs. SLI-Mo 0.41**
	Non-word repetition	USA	Lindgren et al (2009) [47]	ALN-P n = 39 (20Mo/19Fa)<br>ALI-P n = 62 (31Mo/31Fa)	SLI-P n = 70 (35Mo/35Fa)	**ALN-Mo > ALI-Mo > SLI-Mo*****	**ALN-Fa vs. ALI-Fa 0.28**<br>**ALN-Fa vs. SLI-Fa 1.04**<br>**ALI-Fa vs. SLI-Fa 0.59**<br>**ALN-Mo vs. ALI-Mo 0.51**<br>**ALN-Mo vs. SLI-Mo 1.28**<br>**ALI-Mo vs. SLI-Mo 0.83**
		USA	Schmidt et al (2008) [10]	ASD-P n = 22 (14Mo/8Fa)	UA n = 22 (14F/8M)	**ASD-P < UA****	**ASD-P vs. UA 0.87**
Nonword Memory Test	Raw score	Australia	Bishop et al (2004b) [53]	ASD-P = 142 (77Mo/65Fa)	N-P n = 96 (57Mo/39Fa)	**n.s.**	**ASD-Fa vs. N-Fa 0.04**<br>**ASD-Mo vs. N-Mo 0.02**
Nonsense Passage Reading Test	Total score	Australia	Bishop et al (2004b) [53]	ASD-P = 145 (80Mo/65Fa)	N-P n = 96 (57Mo/39Fa)	**n.s.**	**ASD-Fa vs. N-Fa 0.04**<br>**ASD-Mo vs. N-Mo 0.42**
Nepsy Test Battery - Repetition of Nonsense Words Subtest	Raw score	UK	Whitehouse et al (2007) [23]	ASD-P n = 30 (20Mo/10Fa)	SLI-P n= 30 (22Mo/8Fa)<br>TD-P n = 30 (23Mo/7Fa)	**ASD-P > SLI-P****	**ASD-P vs. SLI-P 0.88**<br>**ASD-P vs. TD-P 0.04**
Battery of verbal tasks (emphasis on orthographic and phonological abilities)	Composite Verbal Score	France	Plumet et al (1995) [54]	ASD-P n = 47 (25Mo/22Fa)	DS-P n = 44 (23Mo/21Fa)	**n.s.**	**ASD-P vs. DS-P 0.22**

Reading Skills	Rapid Automized Naming (RAN)							
	Number	Time to complete	USA	Piven & Palmer (1997) [46]	MPX-P n= 48 (25Mo/23Fa)	DS-P n = 60 (30Mo/30Fa)	**n.s.**	**MPX-P vs. DS-P 0.19**
	Letter	Time to complete	USA	Piven & Palmer (1997) [46]	MPX-P n= 48 (25Mo/23Fa)	DS-P n = 60 (30Mo/30Fa)	**n.s.**	**MPX-P vs. DS-P 0.17**
	Colour	Time to complete	USA	Piven & Palmer (1997) [46]	MPX-P n= 48 (25Mo/23Fa)	DS-P n = 60 (30Mo/30Fa)	**MPX-P > DS-P***	**MPX-P vs. DS-P 0.72**
	Object	Time to complete	USA	Piven & Palmer (1997) [46]	MPX-P n= 48 (25Mo/23Fa)	DS-P n = 60 (30Mo/30Fa)	**MPX-P > DS-P***	**MPX-P vs. DS-P 0.58**
	Color/Object combined	Time to complete	USA	Losh et al (2010) [55]	ASD-P n = 301	TD-P/DS-P n = 87	**ASD-P > TD-P/DS-P****	
	Woodcock-Johnson Psycho-Educational Battery - Revised (WJ-R)	Broad reading composite	USA	Lindgren et al (2009) [47]	ALN-P n = 39 (20Mo/19Fa) ALI-P n = 62 (31Mo/31Fa)	SLI-P n = 70 (35Mo/35Fa)	**n.s.**	**ALN-Fa vs. ALI-Fa 0.78<br>ALN-Fa vs. SLI-Fa 2.11<br>ALI-Fa vs. SLI-Fa 1.06<br>ALN-Mo vs. ALI-Mo 0.48<br>ALN-Mo vs. SLI-Mo 1.69<br>ALI-Mo vs. SLI-Mo 1.26**
		Reading skill composite	USA	Lindgren et al (2009) [47]	ALN-P n = 39 (20Mo/19Fa) ALI-P n = 62 (31Mo/31Fa)	SLI-P n = 70 (35Mo/35Fa)	**n.s.**	**ALN-Fa vs. ALI-Fa 0.69<br>ALN-Fa vs. SLI-Fa 1.84<br>ALI-Fa vs. SLI-Fa 1.00<br>ALN-Mo vs. ALI-Mo 0.40<br>ALN-Mo vs. SLI-Mo 1.67<br>ALI-Mo vs. SLI-Mo 1.24**
		Nonsense Word Reading – Reading age	USA	Folstein et al (1999) [30]	ASD-P n = 166	DS-P n = 75	**ASD-P < DS-P***	**ASD-P vs. DS-P 0.48**
		Nonsense Word Reading – Reading grade	USA	Folstein et al (1999) [30]	ASD-P n = 166	DS-P n = 75	**ASD-P < DS-P***	**ASD-P vs. DS-P 0.40**
		Dictation	USA	Lindgren et al (2009) [47]	ALN-P n = 39 (20Mo/19Fa) ALI-P n = 62 (31Mo/31Fa)	SLI-P n = 70 (35Mo/35Fa)	**ALN-Mo > ALI-Mo > SLI-Mo*****	**ALN-Fa vs. ALI-Fa 0.66<br>ALN-Fa vs. SLI-Fa 1.65<br>ALI-Fa vs. SLI-Fa 0.87<br>ALN-Mo vs. ALI-Mo 0.36**

							**ALN-Mo vs. SLI-Mo 1.26**<br>**ALI-Mo vs. SLI-Mo 0.99**
		USA	Piven & Palmer (1997) [46]	MPX-P n= 48 (25Mo/23Fa)	DS-P n = 60 (30Mo/30Fa)	**n.s.**	**MPX-P vs. DS-P 0.17**
	Passage comprehension	USA	Lindgren et al (2009) [47]	ALN-P n = 39 (20Mo/19Fa)<br>ALI-P n = 62 (31Mo/31Fa)	SLI-P n = 70 (35Mo/35Fa)	**ALN-Mo > ALI-Mo > SLI-Mo***	**ALN-Fa vs. ALI-Fa 0.59**<br>**ALN-Fa vs. SLI-Fa 1.54**<br>**ALI-Fa vs. SLI-Fa 0.79**<br>**ALN-Mo vs. ALI-Mo 0.50**<br>**ALN-Mo vs. SLI-Mo 1.46**<br>**ALI-Mo vs. SLI-Mo 1.01**
		USA	Piven & Palmer (1997) [46]	MPX-P n= 48 (25Mo/23Fa)	DS-P n = 60 (30Mo/30Fa)	**MPX-P < DS-P***	**MPX-P vs. DS-P 0.45**
	Word Attack	USA	Lindgren et al (2009) [47]	ALN-P n = 39 (20Mo/19Fa)<br>ALI-P n = 62 (31Mo/31Fa)	SLI-P n = 70 (35Mo/35Fa)	**n.s.**	**ALN-Fa vs. ALI-Fa 0.48**<br>**ALN-Fa vs. SLI-Fa 1.30**<br>**ALI-Fa vs. SLI-Fa 0.75**<br>**ALN-Mo vs. ALI-Mo 0.31**<br>**ALN-Mo vs. SLI-Mo 1.35**<br>**ALI-Mo vs. SLI-Mo 1.06**
		USA	Piven & Palmer (1997) [47]	MPX-P n= 48 (25Mo/23Fa)	DS –P n = 60 (30Mo/30Fa)	**n.s.**	**MPX-P vs. DS-P 0.09**
	Letter Word	USA	Piven & Palmer (1997) [46]	MPX-P n= 48 (25Mo/23Fa)	DS n = 60 (30Mo/30Fa)	**n.s.**	**MPX-P vs. DS-P 0.24**
Gray Oral Reading Test (GORT)	Comprehension	USA	Folstein et al (1999) [30]	ASD-P n = 166	DS-P n = 75	**n.s.**	**ASD-P vs. DS-P 0.12**
	Passage	USA	Folstein et al (1999) [30]	ASD-P n = 166	DS-P n = 75	**n.s.**	**ASD-P vs. DS-P 0.21**
	Reading age	UK	Fombonne et al (1997) [44]	ASD-P n = 160 (86Mo/74Fa)	DS-P n = 42 (23Mo/19Fa)	**n.s.**	**ASD-P vs. DS-P 0.36**
Edinburgh Reading Test (ERT)	Reading age	UK	Fombonne et al (1997) [44]	ASD-P n = 160 (86Mo/74Fa)	DS-P n = 42 (23Mo/19Fa)	**n.s.**	**ASD-P vs. DS-P 0**
National Adult	Error score	UK	Baron-Cohen &	AS-P n = 30	TD-P n = 30	**n.s.**	**ASD-P vs, N-P 0.20**

	Reading Test (NART)			Hammer (1997) [7]	(15Mo/15Fa)	(15Mo/15Fa)		
			UK	Fombonne et al (1997) [44]	ASD-P n = 160 (86Mo/74Fa)	DS-P n = 42 (23Mo/19Fa)	**ASD-P < DS-P***	**ASD-P vs. DS-P 0.44**
	Test of Word Reading Efficiency							
	Sight Word Efficiency Subtest (real words)	Standard score	UK	Whitehouse et al (2007) [23]	ASD-P n = 30 (20Mo/10Fa)	SLI-P n= 30 (22Mo/8Fa)<br>TD-P n = 30 (23Mo/7Fa)	**n.s.**	**ASD-P vs. SLI-P 0.18**<br>**ASD-P vs. TD-P 0.03**
	Phonemic Decoding Efficiency Subtest (nonsense words)	Standard score	UK	Whitehouse et al (2007) [23]	ASD-P n = 30 (20Mo/10Fa)	SLI-P n= 30 (22Mo/8Fa)<br>TD-P n = 30 (23Mo/7Fa)	**ASD-P > SLI-P***	**ASD-P vs. SLI-P 0.62**<br>**ASD-P vs. TD-P 0.24**
	Reading History Questionnaire (RHQ)	Reading difficulties	USA	Schmidt et al (2008) [10]	ASD-P n = 22 (14Mo/8Fa)	UA n = 22 (14F/8M)	**n.s.**	**ASD-P vs. UA 0.34**
Spelling abilities	Schonell Spelling Test (SST)	Total words correct	USA	Folstein et al (1999) [30]	ASD-P n = 166	DS-P n = 75	**n.s.**	**ASD-P vs. DS-P 0.13**
			UK	Fombonne et al (1997) [44]	ASD-P n = 160 (86Mo/74Fa)	DS-P n = 42 (23Mo/19Fa)	**ASD-P > DS-P****	**ASD-P vs. DS-P 0.62**
		Spelling age	USA	Folstein et al (1999) [30]	ASD-P n = 166	DS-P n = 75	**n.s.**	**ASD-P vs. DS-P 0.02**
	Speeded Dictation task	Raw Score	UK	Whitehouse et al (2007) [23]	ASD-P n = 30 (20Mo/10Fa)	SLI-P n= 30 (22Mo/8Fa)<br>TD-P n = 30 (23Mo/7Fa)	**n.s.**	

Oromotor Functioning	Oromotor Sequencing Subtest - NEPSY Test Battery	Raw score	UK	Whitehouse et al (2007) [23]	ASD-P n = 30 (20Mo/10Fa)	SLI-P n= 30 (22Mo/8Fa) TD-P n = 30 (23Mo/7Fa)	**ASD-P > SLI-P****	**ASD-P vs. SLI-P 0.54 ASD-P vs. TD-P 0.43**
**Social Cognition**	Reading the Mind in Eyes Test	Accuracy	UK	Baron-Cohen & Hammer (1997) [7]	AS-P n = 30 (15Mo/15Fa)	TD-P n = 30 (15Mo/15Fa)	**ASD-Fa < N-Fa** ASD-Mo < N-Mo*****	**ASD-Fa vs. N-Fa 0.99 ASD-Mo vs. N-Mo 1.51**
Theory of Mind			USA	Losh & Piven (2007) [46]	ASD-P n = 48 (25Mo/23Fa) BAP(+) A*loof* n = 13 BAP(+) R*igid* n = 11 BAP(-) n = 24	TD/DS-P n = 22 TD-P n = 16 DS-P n = 6	**BAP(+) A*loof* < TD/DS-P** BAP(+) Aloof < BAP(-)****	**BAP (+) Aloof vs. TD/DS-P 1.51 BAP (+) Aloof vs. BAP(-) 1.49 BAP (+) Aloof vs. BAP (+) Rigid 1.48**
			USA	Losh et al (2009) [56]	ASD-P n= 83 BAP(+) *Social* n = 22 BAP(+) *Rigid/Perfectionistic* n = 34 BAP(+) *Social & Rigid* n = 13 (5Mo/8Fa) BAP(-) n = 40	TD-P n = 32 (19Mo/13Fa)	**ASD-P < TD-P* BAP(+) Social< BAP(-)*** BAP(+) Social < TD-P*****	
			Turkey	Gocken et al (2009) [57]	ASD-P n = 76 (38Mo/38Fa)	N-P n = 41 (21Mo/20Fa)	**n.s.**	**ASD-P vs. N-P 0.43**
			Iran	Tajmirriyahi et al (2013) [58]	ASD-P n = 48 (38Mo/10Fa)	DS-P n = 31 (25Mo/6Fa) TD-P n = 30 (23Mo/7Fa)	**n.s.**	**ASD-P vs. DS-P 0.33 ASD-P vs. TD-P 0.03**
	**The Faces Test (mental state decoding)**	Accuracy	Iran	Gocken et al (2009) [57]	ASD-P n = 76 (38Mo/38Fa)	N-P n = 41 (21Mo/20Fa)	**n.s.**	**ASD-P vs. N-P 0.23**
	**Reading the Mind in the Voice Test**	Accuracy	Iran	Tajmirriyahi et al (2013) [58]	ASD-P n = 48 (38Mo/10Fa)	DS-P n = 31 (25Mo/6Fa) TD-P n = 30 (23Mo/7Fa)	**ASD-P < DS-P**, TD-P*****	**ASD-P vs. DS-P 0.63 ASD-P vs. TD-P 0.98**

	**False belief tasks: *'Smarties task'; Sally-Anne task; Unexpected transfer test***	No. of tasks passed	Italy	Di Michele et al (2007) [8]	ASD n = 46	DS-P n=14<br>TD-P n=12	**ASD-P < TD-P*****<br>**ASD-P < DS-P*****	
	**Unexpected Outcomes Test (UOT)**	Total score	Turkey	Gocken et al (2009) [57]	ASD-P n = 76 (38Mo/38Fa)	N-P n = 41 (21Mo/20Fa)	**ASD-P < N-P****	**ASD-P vs. N-P 0.58**
	**The Hinting Task**	Accuracy	Turkey	Gocken et al (2009) [57]	ASD-P n = 76 (38Mo/38Fa)	N-P n = 41 (21Mo/20Fa)	**n.s.**	**ASD-P vs. N-P 0.36**
Empathy	**Empathy Quotient (EQ)**	Mean score	UK	Sucksmith et al (2013) [52]	ASD-P n = 310 (272Mo/38Fa)	UA n = 187 (93M/94F)	**ASD-Fa < UA-M***	**ASD-Fa vs. US-M 0.40**<br>**ASD-Mo vs. UA-F 0.11**
Affect Perception / Emotion Recognition	**Pictures of Facial affect - 'Bubbles' method**	Accuracy	USA	Adolphs et al (2008) [59]	AD-P n = 42 total<br>BAP(+) *Aloof* n = 15 (3Mo/12Fa)<br>BAP(-) n = 27 (20Mo/7Fa)	TD-P n = 20 (8Mo/12Fa)	**n.s.**	
		Reaction Time	USA	Adolphs et al (2008) [59]	AD-P n = 42 total<br>BAP(+) *Aloof* n = 15 (3Mo/12Fa)<br>BAP(-) n = 27 (20Mo/7Fa)	TD-P n = 20 (8Mo/12Fa)	**n.s.**	
		Use of Facial Information<br>*Eyes region*<br>*Mouth region*	USA	Adolphs et al (2008) [59]	AD-P n = 42 total<br>BAP(+) *Aloof* n = 15 (3Mo/12Fa)<br>BAP(-) n = 27 (20Mo/7Fa)	TD-P n = 20 (8Mo/12Fa)	**BAP(+) <BAP(-) <TD-P*****<br>**BAP(+) >BAP(-) >TD-P*****	
	**Penn Emotion**	Time for correct	Brazil	das Neves et al	ASD-P n = 40	UA n = 41	**ASD-P > UA*****	**ASD-P vs. UA 1.09**

**Recognition Test (ER40)**	answers		(2011) [60]	(30Mo/10Fa)	(28F/13M)		
	Accuracy	Brazil	das Neves et al (2011) [60]	ASD-P n = 40 (30Mo/10Fa)	UA n = 41 (28F/13M)	**ASD-P < UA*** **	**ASD-P vs. UA 0.76**
	Female faces	Brazil	das Neves et al (2011) [60]	ASD-P n = 40 (30Mo/10Fa)	UA n = 41 (28F/13M)	**ASD-P < UA*** **	**ASD-P vs. UA 0.66**
	Male faces	Brazil	das Neves et al (2011) [60]	ASD-P n = 40 (30Mo/10Fa)	UA n = 41 (28F/13M)	**ASD-P < UA***	**ASD-P vs. UA 0.63**
	Mild emotions	Brazil	das Neves et al (2011) [60]	ASD-P n = 40 (30Mo/10Fa)	UA n = 41 (28F/13M)	**ASD-P < UA***	**ASD-P vs. UA 0.61**
	Extreme emotions	Brazil	das Neves et al (2011) [60]	ASD-P n = 40 (30Mo/10Fa)	UA n = 41 (28F/13M)	**ASD-P < UA***	**ASD-P vs. UA 0.54**
**Facial Affect Recognition Test (pictures by Ekman & Friesen)**	Expected answers	Germany / Austria / Switzerland	Bölte & Poustka (2003) [49]	ASD SPX-P n = 54 (26Mo/ 28Fa) ASD MPX-P n = 28 (16Mo/12Fa)	Sch SPX-P n = 31 (18Mo/ 13Fa) Sch MPX-P n = 4 (2Mo/2Fa) UA n = 22 (11F/11M)	**n.s.**	**ASD MPX-P vs. Sch MPX-P 0.54 ASD MPX vs. Sch SPX-P 0.36 ASD MPX-P vs. UA 2.06 ASD SPX-P vs. Sch MPX-P 1.39 ASD SPX-P vs. Sch SPX-P 0.57 ASD SPX-P vs. UA 0.32**
**Emotion Recognition Test - using set of photographs from Ekman & Friesen's (1976) 'Photos of Facial Affect'**	Happiness	Turkey	Kadak et al (2014) [21]	ASD-P n=72 (36Mo/36Fa)	TD-P n=38 (19Mo/19Fa)	**ASD-P < TD-P***	**ASD-P vs. TD-P 0.05**
	Sadness	Turkey	Kadak et al (2014) [21]	ASD-P n=72 (36Mo/36Fa)	TD-P n=38 (19Mo/19Fa)	**n.s.**	**ASD-P vs. TD-P 0.07**
	Fearful	Turkey	Kadak et al (2014) [21]	ASD-P n=72 (36Mo/36Fa)	TD-P n=38 (19Mo/19Fa)	**n.s.**	**ASD-P vs. TD-P 0.19**
	Disgusted	Turkey	Kadak et al (2014) [21]	ASD-P n=72 (36Mo/36Fa)	TD-P n=38 (19Mo/19Fa)	**n.s.**	**ASD-P vs. TD-P 0.28**
	Angry	Turkey	Kadak et al (2014) [21]	ASD-P n=72 (36Mo/36Fa)	TD-P n=38 (19Mo/19Fa)	**n.s.**	**ASD-P vs. TD-P 0.09**
	Surprised	Turkey	Kadak et al (2014) [21]	ASD-P n=72 (36Mo/36Fa)	TD-P n=38 (19Mo/19Fa)	**ASD-P < TD-P***	**ASD-P vs. TD-P 0.40**
	Neutral	Turkey	Kadak et al	ASD-P n=72	TD-P n=38	**ASD-P < TD-P***	**ASD-P vs. TD-P 0.50**

			(2014) [21]	(36Mo/36Fa)	(19Mo/19Fa)		
**Karolinska Directed Emotional Faces task (KDEF)**	Mean accuracy p/emotion	UK	Sucksmith et al (2013) [52]	ASD-P n = 297 (261Mo/36Fa)	UA n = 184 (92M/92F)	**n.s.**	**ASD-Fa vs. UA-M 0.12**<br>**ASD-Mo vs. UA-F 0.08**
	Overall mean accuracy adjusted response time p/emotion	UK	Sucksmith et al (2013) [52]	ASD-P n = 297 (261Mo/36Fa)	UA n = 184 (92M/92F)	**n.s.**	**ASD-Fa vs. UA-M 0.30**<br>**ASD-Mo vs. UA-F 0.20**
**Point light basic emotions**							
Positive emotions	Accuracy	USA	Losh et al (2009) [56]	ASD-P n= 83<br>BAP(+) *Social* n = 22<br>BAP(+) *Rigid/Perfectionistic* n = 34<br>BAP(+) *Social & Rigid* n = 13 (5Mo/8Fa)<br>BAP(-) n = 40	TD-P n = 32 (19Mo/13Fa)	**ASD-P < TD-P****	
Negative emotions	Accuracy	USA	Losh et al (2009) [56]	ASD-P n= 83<br>BAP(+) *Social* n = 22<br>BAP(+) *Rigid/Perfectionistic* n = 34<br>BAP(+) *Social & Rigid* n = 13 (5Mo/8Fa)<br>BAP(-) n = 40	TD-P n = 32 (19Mo/13Fa)	**n.s.**	
**Point light trustworthiness**							
Positive stimuli	Accuracy	USA	Losh et al (2009) [56]	ASD-P n= 83<br>BAP(+) *Social* n = 22<br>BAP(+) *Rigid/Perfectionistic* n = 34<br>BAP(+) *Social & Rigid* n = 13 (5Mo/8Fa)<br>BAP(-) n = 40	TD-P n = 32 (19Mo/13Fa)	**ASD-P <TD-P****<br>**BAP(+) Social < BAP (-)****	

Negative stimuli	Accuracy	USA	Losh et al (2009) [56]	ASD-P n= 83<br>BAP(+) *Social* n = 22<br>BAP(+) *Rigid/Perfectionistic* n = 34<br>BAP(+) *Social & Rigid* n = 13 (5Mo/8Fa)<br>BAP(-) n = 40	TD-P n = 32 (19Mo/13Fa)	**BAP(+) Social > BAP(-)***
**Trustworthiness of faces**						
Positive faces	Judgment of trustworthiness	USA	Losh et al (2009) [56]	ASD-P n= 83<br>BAP(+) *Social* n = 22<br>BAP(+) *Rigid/Perfectionistic* n = 34<br>BAP(+) *Social & Rigid* n = 13 (5Mo/8Fa)<br>BAP(-) n = 40	TD-P n = 32 (19Mo/13Fa)	**ASD-P > TD-P*****
Negative faces	Judgment of trustworthiness	USA	Losh et al (2009) [56]	ASD-P n= 83<br>BAP(+) *Social* n = 22<br>BAP(+) *Rigid/Perfectionistic* n = 34<br>BAP(+) *Social & Rigid* n = 13 (5Mo/8Fa)<br>BAP(-) n = 40	TD-P n = 32 (19Mo/13Fa)	**BAP(+) Social < BAP(-), TD-P*****
**The Morphed faces task**						
**Happy**	Accuracy	USA	Losh et al (2009) [56]	ASD-P n= 83<br>BAP(+) *Social* n = 22<br>BAP(+) *Rigid/Perfectionistic* n = 34<br>BAP(+) *Social & Rigid* n = 13 (5Mo/8Fa)<br>BAP(-) n = 40	TD-P n = 32 (19Mo/13Fa)	**n.s.**

Low morphedness	Accuracy	USA	Losh et al (2009) [56]	ASD-P n= 83<br>BAP(+) *Social* n = 22<br>BAP(+) *Rigid/Perfectionistic* n = 34<br>BAP(+) *Social & Rigid* n = 13 (5Mo/8Fa)<br>BAP(-) n = 40	TD-P n = 32 (19Mo/13Fa)	**n.s.**
High morphedness	Accuracy	USA	Losh et al (2009) [56]	ASD-P n= 83<br>BAP(+) *Social* n = 22<br>BAP(+) *Rigid/Perfectionistic* n = 34<br>BAP(+) *Social & Rigid* n = 13 (5Mo/8Fa)<br>BAP(-) n = 40	TD-P n = 32 (19Mo/13Fa)	**n.s.**
**Sad**	Accuracy	USA	Losh et al (2009) [56]	ASD-P n= 83<br>BAP(+) *Social* n = 22<br>BAP(+) *Rigid/Perfectionistic* n = 34<br>BAP(+) *Social & Rigid* n = 13 (5Mo/8Fa)<br>BAP(-) n = 40	TD-P n = 32 (19Mo/13Fa)	**n.s.**
Low morphedness	Accuracy	USA	Losh et al (2009) [56]	ASD-P n= 83<br>BAP(+) *Social* n = 22<br>BAP(+) *Rigid/Perfectionistic* n = 34<br>BAP(+) *Social & Rigid* n = 13 (5Mo/8Fa)<br>BAP(-) n = 40	TD-P n = 32 (19Mo/13Fa)	**n.s.**
High morphedness	Accuracy	USA	Losh et al (2009) [56]	ASD-P n= 83<br>BAP(+) *Social* n = 22<br>BAP(+) *Rigid/Perfectionistic* n = 34	TD-P n = 32 (19Mo/13Fa)	**n.s.**

				BAP(+) *Social & Rigid* n = 13 (5Mo/8Fa)<br>BAP(-) n = 40		
**Afraid**	Accuracy	USA	Losh et al (2009) [56]	ASD-P n= 83<br>BAP(+) *Social* n = 22<br>BAP(+) *Rigid/Perfectionistic* n = 34<br>BAP(+) *Social & Rigid* n = 13 (5Mo/8Fa)<br>BAP(-) n = 40	TD-P n = 32 (19Mo/13Fa)	**ASD-P < TD-P****
Low morphedness	Accuracy	USA	Losh et al (2009) [56]	ASD-P n= 83<br>BAP(+) *Social* n = 22<br>BAP(+) *Rigid/Perfectionistic* n = 34<br>BAP(+) *Social & Rigid* n = 13 (5Mo/8Fa)<br>BAP(-) n = 40	TD-P n = 32 (19Mo/13Fa)	**BAP(+) Social < TD-P***
High morphedness	Accuracy	USA	Losh et al (2009) [56]	ASD-P n= 83<br>BAP(+) *Social* n = 22<br>BAP(+) *Rigid/Perfectionistic* n = 34<br>BAP(+) *Social & Rigid* n = 13 (5Mo/8Fa)<br>BAP(-) n = 40	TD-P n = 32 (19Mo/13Fa)	**n.s.**
**The Movie Stills task**<br><br>***Without faces***	Accuracy	USA	Losh et al (2009) [56]	ASD-P n= 83<br>BAP(+) *Social* n = 22<br>BAP(+) *Rigid/Perfectionistic* n = 34<br>BAP(+) *Social & Rigid* n = 13 (5Mo/8Fa)	TD-P n = 32 (19Mo/13Fa)	**n.s.**

				BAP(-) n = 40		
**Sad**	Accuracy	USA	Losh et al (2009) [56]	ASD-P n= 83<br>BAP(+) *Social* n = 22<br>BAP(+) *Rigid/Perfectionistic* n = 34<br>BAP(+) *Social & Rigid* n = 13 (5Mo/8Fa)<br>BAP(-) n = 40	TD-P n = 32 (19Mo/13Fa)	**ASD-P < TD-P****
**Angry**	Accuracy	USA	Losh et al (2009) [56]	ASD-P n= 83<br>BAP(+) *Social* n = 22<br>BAP(+) *Rigid/Perfectionistic* n = 34<br>BAP(+) *Social & Rigid* n = 13 (5Mo/8Fa)<br>BAP(-) n = 40	TD-P n = 32 (19Mo/13Fa)	**ASD-P < TD-P***
**Afraid**	Accuracy	USA	Losh et al (2009) [56]	ASD-P n= 83<br>BAP(+) *Social* n = 22<br>BAP(+) *Rigid/Perfectionistic* n = 34<br>BAP(+) *Social & Rigid* n = 13 (5Mo/8Fa)<br>BAP(-) n = 40	TD-P n = 32 (19Mo/13Fa)	**ASD-P > TD-P*****
With faces	Accuracy	USA	Losh et al (2009) [56]	ASD-P n= 83<br>BAP(+) *Social* n = 22<br>BAP(+) *Rigid/Perfectionistic* n = 34<br>BAP(+) *Social & Rigid* n = 13 (5Mo/8Fa)<br>BAP(-) n = 40	TD-P n = 32 (19Mo/13Fa)	**BAP(+) Social < BAP(-), TD-P***
**Sad**	Accuracy	USA	Losh et al (2009)[56]	ASD-P n= 83<br>BAP(+) *Social* n = 22<br>BAP(+)	TD-P n = 32 (19Mo/13Fa)	**n.s.**

				Rigid/Perfectionistic n = 34<br>BAP(+) *Social & Rigid* n = 13 (5Mo/8Fa)<br>BAP(-) n = 40		
**Angry**	Accuracy	USA	Losh et al (2009) [56]	ASD-P n= 83<br>BAP(+) *Social* n = 22<br>BAP(+) *Rigid/Perfectionistic* n = 34<br>BAP(+) *Social & Rigid* n = 13 (5Mo/8Fa)<br>BAP(-) n = 40	TD-P n = 32 (19Mo/13Fa)	**ASD-P < TD-P****
**Afraid**	Accuracy	USA	Losh et al (2009) [56]	ASD-P n= 83<br>BAP(+) *Social* n = 22<br>BAP(+) *Rigid/Perfectionistic* n = 34<br>BAP(+) *Social & Rigid* n = 13 (5Mo/8Fa)<br>BAP(-) n = 40	TD-P n = 32 (19Mo/13Fa)	**n.s.**
**Schematic Line Drawings (emotional labeling of facial patterns)**						
**Anger**		Italy	Palermo et al (2006) [61]	ASD-P n= 40 (20Mo/20Fa)	TD-P n= 40 (20Mo/20Fa)	**n.s**
**Happiness**		Italy	Palermo et al (2006) [61]	ASD-P n= 40 (20Mo/20Fa)	TD-P n= 40 (20Mo/20Fa)	**n.s.**
**Sadness**		Italy	Palermo et al (2006) [61]	ASD-P n= 40 (20Mo/20Fa)	TD-P n= 40 (20Mo/20Fa)	**ASD-P < TD-P****
**Surprise**		Italy	Palermo et al (2006) [61]	ASD-P n= 40 (20Mo/20Fa)	TD-P n= 40 (20Mo/20Fa)	**n.s.**

	**Disgust**		Italy	Palermo et al (2006) [61]	ASD-P n= 40 (20Mo/20Fa)	TD-P n= 40 (20Mo/20Fa)	**ASD-P < TD-P****	
	**Overall recognition**		Italy	Palermo et al (2006) [61]	ASD-P n= 40 (20Mo/20Fa)	TD-P n= 40 (20Mo/20Fa)	**ASD-P < TD-P****	
	**Emotion Matching Task**		USA	Smalley & Asarnow (1990) [45]	ASD-P n = 15	TD-P n = 12	**n.s.**	**ASD-P vs. TD-P 0.06**
	**Emotion Labeling Task**		USA	Smalley & Asarnow (1990) [45]	ASD-P n = 15	TD-P n = 12	**n.s.**	**ASD-P vs. TD-P 0.19**
**Executive Function** ***Set-Shifiting***	**Intradimensional - Extradimensional set-shifting task (IDED)**	Perseveration (EDS Stage)	Australia	Wong et al (2006)[62]	ASD-P n = 145 (80Mo/65Fa)	TD-P n = 96 (57Mo/39Fa)	**n.s.**	
		Learned irrelevance (EDS Stage)	Australia	Wong et al (2006) [62]	ASD-P n = 145 (80Mo/65Fa)	TD-P n = 96 (57Mo/39Fa)	**ASD-P > TD-P*** **ASD-Fa > TD-Fa***	**ASD-Fa vs. TD-Fa 0.52**
		Trials to criterion (EDS Stage)	France	Hughes et al (1997) [63]	ASD-P n=40 (20Mo/20Fa)	LD-P n=40 (22Mo/18Fa) UA n=36 (18M, 15F)	**ASD-P > LD-P**** **ASD-P > UA*****	**ASD-P vs. LD-P 0.69** **ASD-P vs. UA 0.83**
		Errors to criterion (EDS Stage)	France	Hughes et al (1997) [63]	ASD-P n=40 (20Mo/20Fa)	LD-P n=40 (22Mo/18Fa) UA n=36 (18M, 15F)	**ASD-P > LD-P*** **ASD-P > UA****	**ASD-P vs. LD-P 0.64** **ASD-P vs. UA 0.70**
	**Wisconsin Card Sorting Test (WCST)**	Perservative errors	Germany	Bölte & Poustka (2006) [50]	ASD-P n=62 (33Mo/29Fa)	EOS-P n = 36 (20Mo/16Fa) MR-P n = 30 (16Mo/14Fa)	**n.s.**	**ASD-P vs. EOS-P 0.06** **ASD-P vs. MR-P 0.18**
	**Trail Making Test (A & B)**	Total time to complete	Germany	Bölte & Poustka (2006) [50]	ASD-P n=62 (33Mo/29Fa)	EOS-P n = 36 (20Mo/16Fa) MR-P n = 30 (16Mo/14Fa)	**n.s.**	**ASD-P vs. EOS-P 0.38** **ASD-P vs. MR-P 0.13**

			USA	Losh et al (2009) [56]	ASD-P n= 83<br>BAP(+) *Social* n = 22<br>BAP(+) *Rigid/Perfectionistic* n = 34<br>BAP(+) *Social & Rigid* n = 13 (5Mo/8Fa)<br>BAP(-) n = 40	TD-P n = 32 (19Mo/13Fa)	**n.s.**	
Planning	**Tower of London (ToL)**	Number of extra moves	France	Hughes et al (1997) [63]	ASD-P n=40 (20Mo/20Fa)	LD-P n=40 (22Mo/18Fa)<br>UA n=36 (18M, 15F)	**ASD-P > UA***	**ASD-P vs. LD-P 0.41**<br>**ASD-P vs. UA 0.93**
		Adjusted extra moves score	Australia	Wong et al (2006) [62]	ASD-P n = 145 (80Mo/65Fa)	TD-P n = 96 (57Mo/39Fa)	**n.s.**	**ASD-P vs. TD-P 0.07**
		Rule violations	Australia	Wong et al (2006) [62]	ASD-P n = 145 (80Mo/65Fa)	TD-P n = 96 (57Mo/39Fa)	**n.s.**	
		Solutions correct	France	Hughes et al (1997) [63]	ASD-P n=40 (20Mo/20Fa)	LD-P n=40 (22Mo/18Fa)<br>UA n=36 (18M, 15F)	**ASD-P < UA*****	**ASD-P vs. LD-P 0.34**<br>**ASD-P vs. UA 0.93**
	**Tower of Hanoi (ToH)**	Total time to complete	Germany	Bölte & Poustka (2006) [50]<br>*4 ring version*	ASD-P n=62 (33Mo/29Fa)	EOS-P n = 36 (20Mo/16Fa)<br>MR-P n = 30 (16Mo/14Fa)	**n.s.**	**ASD-P vs. EOS-P 0.45**<br>**ASD-P vs. MR-P 0.01**
			USA	Losh et al (2009) [56]	ASD-P n= 83<br>BAP(+) *Social* n = 22<br>BAP(+) *Rigid/Perfectionistic* n = 34<br>BAP(+) *Social & Rigid* n = 13 (5Mo/8Fa)<br>BAP(-) n = 40	TD-P n = 32 (19Mo/13Fa)	**n.s.**	
		Planning efficiency score	USA	Piven & Palmer (1997) [46]	MPX-P n= 48 (25Mo/23Fa)	DS-P n = 60 (30Mo/30Fa)	*3 ring version*<br>**MPX-P < DS-P***	*3 ring version*<br>**MPX-P vs. DS-P 0.40**

							4 ring version<br>**MPX-P < DS-P***	*4 ring version*<br>**MPX-P vs. DS-P 0.48**
			USA	Losh et al (2009) [56]	ASD-P n= 83<br>BAP(+) *Social* n = 22<br>BAP(+) *Rigid/Perfectionistic* n = 34<br>BAP(+) *Social & Rigid* n = 13 (5Mo/8Fa)<br>BAP(-) n = 40	TD-P n = 32 (19Mo/13Fa)	**n.s.**	
Generativity / Ideational Fluency	**Pattern Meanings**	Overall response generativity	Australia	Wong et al (2006) [62]	ASD-P n = 145 (80Mo/65Fa)	TD-P n = 96 (57Mo/39Fa)	**ASD-P < TD-P*****	**ASD-P vs. TD-P 0.51**
Spatial Working Memory / Inhibition	**Visual Search Test**	Between search errors	France	Hughes et al (1997) [63]	ASD-P n=40 (20Mo/20Fa)	LD-P n=40 (22Mo/18Fa)<br>UA n=36 (18M, 15F)	**ASD-P > UA***<br>**ASD-Fa > UA-M***	**ASD-P vs. LD-P 0.27**<br>**ASD-P vs. UA 0.95**
		Within search errors	France	Hughes et al (1997) [63]	ASD-P n=40 (20Mo/20Fa)	LD-P n=40 (22Mo/18Fa)<br>UA n=36 (18M, 15F)	**n.s.**	
	**The Delayed Oculomotor Task**<br><br>**(Eye movement abnormality)**	Percent premature saccades<br>1s delay<br>3s delay	USA	Koczat et al (2002) [77]	ASD-P n = 11 (7Mo/4Fa)	UA n = 17 (8F/9M)	**n.s.**	**ASD-P vs. UA 0.18**<br>**ASD-P vs. UA 0.55**
		Latency remembered saccades<br>1s delay<br>3s delay	USA	Koczat et al (2002) [77]	ASD-P n = 11 (7Mo/4Fa)	UA n = 17 (8F/9M)	**n.s.**	**ASD-P vs. UA 0.41**<br>**ASD-P vs. UA 0.04**
		Spatial error of remembered saccades (accuracy)<br>1s delay<br>3s delay	USA	Koczat et al (2002) [77]	ASD-P n = 11 (7Mo/4Fa)	UA n = 17 (8F/9M)	**ASD-P < UA****	**ASD-P vs. UA 1.27**<br>**ASD-P vs. UA 0.68**

	**Response Inhibition and Load (RIL)**	No. of errors	Australia	Wong et al (2006) [62]	ASD-P n = 141	TD-P n = 94	**n.s.**	**ASD-P vs. TD-P 0.28**
		Reaction time for correct responses	Australia	Wong et al (2006) [62]	ASD-P n = 141	TD-P n = 94	**n.s.**	**ASD-P vs. TD-P 0.04**
		Working memory measure	Australia	Wong et al (2006) [62]	ASD-P n = 141	TD-P n = 94	**n.s.**	**ASD-P vs. TD-P 0.08**
Verbal Working Memory	**Auditory Consonant Trigrams (ACT)**	Accuracy	Turkey	Gocken et al (2009) [57]	ASD-P n = 76 (38Mo/38Fa)	N-P n = 41 (21Mo/20Fa)	**ASD-P < N-P****	**ASD-P vs. N-P 0.55**
	**Verbal Fluency (letters KAS in Turkish)**	Accuracy	Turkey	Gocken et al (2009) [57]	ASD-P n = 76 (38Mo/38Fa)	N-P n = 41 (21Mo/20Fa)	**n.s.**	**ASD-P vs. N-P 0.26**
	**Stroop Interference Test**	Interference score	Turkey	Gocken et al (2009) [57]	ASD-P n = 76 (38Mo/38Fa)	N-P n = 41 (21Mo/20Fa)	**n.s.**	**ASD-P vs. N-P 0.2**
**Central Coherence (Local visual processing)** ***Disembedding Performance***	**Embedded Figures Test (EFT)**	Reaction Time	UK	Baron-Cohen & Hammer (1997) [7]	AS-P n = 30 (15Mo/15Fa)	TD-P n = 30 (15Mo/15Fa)	**ASD-Fa > N-Fa**** **ASD-Mo > N-Mo****	**ASD-Fa vs. N-Fa 0.51** **ASD-Mo vs. N-Fa 0.68**
			Germany	Bölte & Poustka (2006) [7]	ASD-P n=62 (33Mo/29Fa)	EOS-P n = 36 (20Mo/16Fa) MR-P n = 30 (16Mo/14Fa)	**ASD-P > EOS-P***** **ASD-P > MR-P***	**ASD-P vs. EOS-P 1.60** **ASD-P vs. MR-P 0.79**
			Netherlands	de Jonge et al (2006) [65]	MPX-P n=51 (26Mo/25Fa)	DS-P n = 54 (28Fa/26Mo)	**n.s.**	**MPX-P vs. DS-P 0.01** **MPX-Fa vs. DS-Fa 0.04** **MPX-Mo vs. DS-Mo 0.09**
			UK	Happé et al (2001) [64]	ASD-P n = 43 (21Mo/22Fa)	DLX-P n = 30 (15Mo/15Fa) TD-P n = 20 (10Mo/10Fa)	**ASD-Fa > DLX-Fa**** **ASD-Fa > TD-Fa****	**ASD-Mo vs. DLX-Mo 0.54** **ASD-Mo vs. TD-Mo 0.64** **ASD-Fa vs. DLX-Fa 1.11** **ASD-Fa vs. TD-Fa 1.09**
			USA	Losh et al	ASD-P n= 83	TD-P n = 32	**n.s.**	

				(2009) [56]	BAP(+) *Social* n = 22<br>BAP(+) *Rigid/Perfectionistic* n = 34<br>BAP(+) *Social & Rigid* n = 13 (5Mo/8Fa)<br>BAP(-) n = 40	(19Mo/13Fa)		
		Accuracy	Netherlands	de Jonge et al (2006) [65]	MPX-P n=51 (26Mo/25Fa)	DS-P n = 54 (28Fa/26Mo)	**n.s.**	**MPX-P vs. DS-P 0.16**<br>**MPX-Fa vs. DS-Fa 0.21**<br>**MPX-Mo vs. DS-Mo 0.11**
			UK	Happé et al (2001) [64]	ASD-P n = 43 (21Mo/22Fa)	DLX-P n = 30 (15Mo/15Fa)<br>TD-P n = 20 (10Mo/10Fa)	**n.s.**	**ASD-Mo vs. DLX-Mo 0.26**<br>**ASD-Mo vs. TD-Mo 0.77**<br>**ASD-Fa vs. DLX-Fa 0.17**<br>**ASD-Fa vs. TD-Fa 0.31**
			USA	Losh et al (2009) [56]	ASD-P n= 83<br>BAP(+) *Social* n = 22<br>BAP(+) *Rigid/Perfectionistic* n = 34<br>BAP(+) *Social & Rigid* n = 13 (5Mo/8Fa)<br>BAP(-) n = 40	TD-P n = 32 (19Mo/13Fa)	**n.s.**	
		No. of incorrect responses	Netherlands	de Jonge et al (2006) [65]	MPX-P n=51 (26Mo/25Fa)	DS-P n = 54 (28Fa/26Mo)	**ASD-Fa < DS-Fa***	**MPX-P vs. DS-P 0.32**<br>**MPX-Fa vs. DS-Fa 0.52**<br>**MPX-Mo vs. DS-Mo 0.18**
	**Titchener Circles Illusion**	No. of errors	UK	Happé et al (2001) [64]	ASD-P n = 43 (21Mo/22Fa)	DLX-P n = 30 (15Mo/15Fa)<br>TD-P n = 20 (10Mo/10Fa)	**ASD-Fa < DLX-Fa***	
Mental Segmentation Ability	**Unsegmented Block Design task (adaptation from Weschler subtest)**	Reaction time	UK	Happé et al (2001) [64]	ASD-P n = 43 (21Mo/22Fa)	DLX-P n = 30 (15Mo/15Fa)<br>TD-P n = 20 (10Mo/10Fa)	**ASD-Fa > TD-Fa***	**ASD-Mo vs. DLX-Mo 0.54**<br>**ASD-Mo vs. TD-Mo 0.24**<br>**ASD-Fa vs. DLX-Fa 0.64**<br>**ASD-Fa vs. TD-Fa 0.84**
			USA	Losh et al (2009) [56]	ASD-P n= 83<br>BAP(+) *Social* n = 22<br>BAP(+) *Rigid/Perfectionistic* n =	TD-P n = 32 (19Mo/13Fa)	**n.s.**	

					34<br>BAP(+) *Social & Rigid* n = 13 (5Mo/8Fa)<br>BAP(-) n = 40			
	**Segmented Block Design task (adaptation from Weschler subtest)**	Reaction time	UK	Happé et al (2001) [64]	ASD-P n = 43 (21Mo/22Fa)	DLX-P n = 30 (15Mo/15Fa)<br>TD-P n = 20 (10Mo/10Fa)	**n.s.**	**ASD-Mo vs. DLX-Mo 0.04**<br>**ASD-Mo vs. TD-Mo 0.63**<br>**ASD-Fa vs. DLX-Fa 0.10**<br>**ASD-Fa vs. TD-Fa 0.17**
			USA	Losh et al (2009) [56]	ASD-P n= 83<br>BAP(+) *Social* n = 22<br>BAP(+) *Rigid/Perfectionistic* n = 34<br>BAP(+) *Social & Rigid* n = 13 (5Mo/8Fa)<br>BAP(-) n = 40	TD-P n = 32 (19Mo/13Fa)	**n.s.**	
	**Block Design task (Weschler subtest)**	Reaction time	Netherlands	Scheeren & Stauder (2008) [24]	ASD-P n= 25 (12Mo/13Fa)	TD-P n= 25 (12Mo/13Fa)	**n.s**	**ASD-Fa vs. TD-Fa 0.19**<br>**ASD-Mo vs. TD-Mo 0.11**
			Germany	Bölte & Poustka (2006) [50]	ASD n=62 (33Mo/29Fa)	EOS-P n = 36 (20Mo/16Fa)<br>MR-P n = 30 (16Mo/14Fa)	**n.s.**	**ASD-P vs. EOS-P 0.33**<br>**ASD-P vs. MR-P 0.52**
	**Block design recontruction task (patterns by Akshoomoff & Stiles)**	Accuracy	Netherlands	de Jonge et al (2009) [66]	MPX-P n = 51 (26Mo/25Fa)	DS-P n = 57 (28Mo/29Fa)	**n.s.**	**MPX-P vs. DS-P 0.16**
		Reconstruction time	Netherlands	de Jonge et al (2009) [66]	MPX-P n = 51 (26Mo/25Fa)	DS-P n = 57 (28Mo/29Fa)	**n.s.**	
		Mean no. of errors	Netherlands	de Jonge et al (2009) [66]	MPX-P n = 51 (26Mo/25Fa)	DS-P n = 57 (28Mo/29Fa)	**n.s.**	**MPX-P vs. DS-P 0.10**
Attentional	**Detection Task (Reaction time task)**	Eyes task (social)	Netherlands	Scheeren & Stauder (2008) [24]	ASD-P n= 25 (12Mo/13Fa)	TD-P n= 25 (12Mo/13Fa)	**ASD-Fa > TD-Fa***	
		Arrows task	Netherlands	Scheeren &	ASD-P n= 25	TD-P n= 25	**n.s**	

Engagement		(non-social)		Stauder (2008) [24]	(12Mo/13Fa)	(12Mo/13Fa)		
Global Sentence Completions	**Sentence Completion task**	Errors (local completions) and long delays	UK	Happé et al (2001) [64]	ASD-P n = 43<br>(21Mo/22Fa)	DLX-P n = 30<br>(15Mo/15Fa)<br>TD-P n = 20<br>(10Mo/10Fa)	**ASD-P > DLX-P > TD-P*****<br>**ASD-Fa > DLX – Fa*****<br>**ASD-Fa > TD-Fa*****<br>**ASD-Mo > DLX-Mo****<br>**ASD-Mo >TD-Mo****	
		Frequency of errors (no. of global responses)	USA	Losh et al (2009) [56]	ASD-P n= 83<br>BAP(+) *Social* n = 22<br>BAP(+) *Rigid/Perfectionistic* n = 34<br>BAP(+) *Social & Rigid* n = 13 (5Mo/8Fa)<br>BAP(-) n = 40	TD-P n = 32<br>(19Mo/13Fa)	**ASD-P > TD-P****	
		Response time	USA	Losh et al (2009) [56]	ASD-P n= 83<br>BAP(+) *Social* n = 22<br>BAP(+) *Rigid/Perfectionistic* n = 34<br>BAP(+) *Social & Rigid* n = 13 (5Mo/8Fa)<br>BAP(-) n = 40	TD-P n = 32<br>(19Mo/13Fa)	**ASD-P > TD-P***<br>**BAP(+) Rigid/Perfectionistic < TD-P****<br>**BAP(-) < TD-P***	
**Visual Processing**<br><br>***Contrast Sensitivity***	**Vistech contrast sensitivity charts**	Mean contrast sensitivity threshold	Netherlands	de Jonge et al (2007) [67]	MPX-P n = 51<br>(26Mo/25Fa)	DS-P n = 52<br>(25Fa/27Mo)	**n.s.**	**MPX-P vs. DS-P 0.55**
Motion Discrimination	**Motion Coherence Task**	Mean motion coherence threshold	Netherlands	de Jonge et al (2007) [67]	MPX-P n = 51<br>(26Mo/25Fa)	DS-P n = 52<br>(25Fa/27Mo)	**n.s.**	**MPX-P vs. DS-P 0.25**
	**Moving Shape Task**	Reaction time	Netherlands	de Jonge et al (2007) [67]	MPX-P n = 51<br>(26Mo/25Fa)	DS-P n = 52<br>(25Fa/27Mo)	**n.s.**	**MPX-P vs. DS-P 0.17**

Form Discrimination	**Form Discrimination (Shape) Task**	Reaction Time	Netherlands	de Jonge et al (2007) [67]	MPX-P n = 51 (26Mo/25Fa)	DS-P n = 52 (25Fa/27Mo)	**n.s.**	**MPX-P vs. DS-P 0.05**

Note: ASD, Autism Spectrum Disorder; BAP, Broad Autism Phenotype; BAP(+), BAP present; BAP (-), BAP absent; P, Parent; Mo, Mother; Fa, Father; M, Male; F, Female; MPX, Multiple incidence autism families; SPX, Single incidence autism families; ALN, Autism without language impairment; ALI, Autism with language impairment; DLX, Dyslexia; DS, Down Syndrome; EOS, Early onset Schizophrenia; LBW, low birth weight; LD, learning difficulties; N, Normative sample; MR, Mental Retardation; Sch SPX, single incidence Schizophrenia families; Sch MPX, multiple incidence Schizophrenia families; SLI, Specific Language Impairment; TD, typically developing; UA, Unaffected adult

*p<0.05; **p<0.01; ***p<0.001

Supplementary Table 3. Other Psychiatric Conditions Endophenotype Matrix – Review of studies of parents of autistic probands.

Domain	Method / Measure	Factors / Subscales	Country	Study	ASD Parent Group Characteristics	Control Group Characteristics	Key Findings in relation to Proband Diagnosis	
							P value	Effect Size (*d*)
**Other Psychiatric Conditions**	**Brief Psychiatric Rating Scale (BPRS)**	Anxiety	Iran	Gocken et al (2009) [57]	ASD-P n = 76 (38Mo/38Fa)	N-P n = 41 (21Mo/20Fa)	**n.s.**	**ASD-P vs. N-P 0.29**
		Depression	Iran	Gocken et al (2009) [57]	ASD-P n = 76 (38Mo/38Fa)	N-P n = 41 (21Mo/20Fa)	**ASD-P > N-P***	**ASD-P vs. N-P 0.44**
	**Personality Style and Disorder Inventory (PSSI)**	Reserved/schizoid	Germany	Bölte et al (2007) [51]	ASD SPX-P n = 87 (48Mo/39Fa) ASD MPX-P n = 38 (21Mo/17Fa)	OCD-P n = 37 (19Mo/18Fa) EOS-P n = 34 (20Mo/14Fa) MR-P n = 27 (15Mo/12Fa)	**SPX-P / MPX-P > OCD-P, EOS-P****	**MPX-P vs. OCD-P 1.07**<br>**MPX-P vs. EOS-P 1.09**<br>**MPX-P vs. MR-P 0.14**<br>**SPX-P vs. OCD-p 1.06**<br>**SPX-P vs. EOS-P 1.18**<br>**SPX-P vs. MR-P 0.15**
		Self-critical/insecure	Germany	Bölte et al (2007) [51]	ASD SPX-P n = 87 (48Mo/39Fa) ASD MPX-P n = 38 (21Mo/17Fa)	OCD-P n = 37 (19Mo/18Fa) EOS-P n = 34 (20Mo/14Fa) MR-P n = 27 (15Mo/12Fa)	**ASD SPX-P /ASD MPX-P > EOS-P****	**MPX-P vs. OCD-P 0.31**<br>**MPX-P vs. EOS-P 1.15**<br>**MPX-P vs. MR-P 0.32**<br>**SPX-P vs. OCD-p 0.02**<br>**SPX-P vs. EOS-P 0.86**<br>**SPX-P vs. MR-P 0.02**
		Critical/negativistic	Germany	Bölte et al (2007) [51]	ASD SPX-P n = 87 (48Mo/39Fa) ASD MPX-P n = 38 (21Mo/17Fa)	OCD-P n = 37 (19Mo/18Fa) EOS-P n = 34 (20Mo/14Fa) MR-P n = 27 (15Mo/12Fa)	**SPX-P /MPX-P > EOS-P****	**MPX-P vs. OCD-P 0.29**<br>**MPX-P vs. EOS-P 0.92**<br>**MPX-P vs. MR-P 0.03**<br>**SPX-P vs. OCD-p 0.17**<br>**SPX-P vs. EOS-P 0.74**<br>**SPX-P vs. MR-P 0.06**
		Spontaneous/borderline	Germany	Bölte et al (2007) [51]	ASD SPX-P n = 87 (48Mo/39Fa) ASD MPX-P n = 38 (21Mo/17Fa)	OCD-P n = 37 (19Mo/18Fa) EOS-P n = 34 (20Mo/14Fa) MR-P n = 27 (15Mo/12Fa)	**MPX-P > EOS-P****	**MPX-P vs. OCD-P 0.18**<br>**MPX-P vs. EOS-P 0.25**<br>**MPX-P vs. MR-P 0.09**<br>**SPX-P vs. OCD-p 0.39**<br>**SPX-P vs. EOS-P 0.72**<br>**SPX-P vs. MR-P 0.04**
		Quiet/depressive	Germany	Bölte et al (2007) [51]	ASD SPX-P n = 87 (48Mo/39Fa) ASD MPX-P n = 38 (21Mo/17Fa)	OCD-P n = 37 (19Mo/18Fa) EOS-P n = 34 (20Mo/14Fa)	**SPX-P /MPX-P > EOS-P****	**MPX-P vs. OCD-P 0.53**<br>**MPX-P vs. EOS-P 1.03**<br>**MPX-P vs. MR-P 0.31**<br>**SPX-P vs. OCD-p 0.35**

					MR-P n = 27 (15Mo/12Fa)		**SPX-P vs. EOS-P 0.87**<br>**SPX-P vs. MR-P 0.16**
**Symptom Checklist-90-Revised (SCL-90-R)**	Depression	Germany	Bölte et al (2007) [51]	ASD SPX-P n = 87 (48Mo/39Fa)<br>ASD MPX-P n = 38 (21Mo/17Fa)	OCD-P n = 37 (19Mo/18Fa)<br>EOS-P n = 34 (20Mo/14Fa)<br>MR-P n = 27 (15Mo/12Fa)	**MPX-P > OCD-P, EOS-P****	**MPX-P vs. OCD-P 0.89**<br>**MPX-P vs. EOS-P 1.06**<br>**MPX-P vs. MR-P 0.66**<br>**SPX-P vs. OCD-p 0.42**<br>**SPX-P vs. EOS-P 0.57**<br>**SPX-P vs. MR-P 0.15**
	Anxiety	Germany	Bölte et al (2007) [51]	ASD SPX-P n = 87 (48Mo/39Fa)<br>ASD MPX-P n = 38 (21Mo/17Fa)	OCD-P n = 37 (19Mo/18Fa)<br>EOS-P n = 34 (20Mo/14Fa)<br>MR-P n = 27 (15Mo/12Fa)	**n.s.**	**MPX-P vs. OCD-P 0.17**<br>**MPX-P vs. EOS-P 0.92**<br>**MPX-P vs. MR-P 0.29**<br>**SPX-P vs. OCD-p 0.01**<br>**SPX-P vs. EOS-P 0.66**<br>**SPX-P vs. MR-P 0.10**
	Phobic-anxiety	Germany	Bölte et al (2007) [51]	ASD SPX-P n = 87 (48Mo/39Fa)<br>ASD MPX-P n = 38 (21Mo/17Fa)	OCD-P n = 37 (19Mo/18Fa)<br>EOS-P n = 34 (20Mo/14Fa)<br>MR-P n = 27 (15Mo/12Fa)	**SPX-P / MPX-P > OCD-P, EOS-P****	**MPX-P vs. OCD-P 0.25**<br>**MPX-P vs. EOS-P 1.33**<br>**MPX-P vs. MR-P 0.45**<br>**SPX-P vs. OCD-p 0**<br>**SPX-P vs. EOS-P 0.91**<br>**SPX-P vs. MR-P 0.19**
	Paranoid ideation	Germany	Bölte et al (2007) [51]	ASD SPX-P n = 87 (48Mo/39Fa)<br>ASD MPX-P n = 38 (21Mo/17Fa)	OCD-P n = 37 (19Mo/18Fa)<br>EOS-P n = 34 (20Mo/14Fa)<br>MR-P n = 27 (15Mo/12Fa)	**MPX-P > EOS-P****	**MPX-P vs. OCD-P 0.44**<br>**MPX-P vs. EOS-P 0.93**<br>**MPX-P vs. MR-P 0.57**<br>**SPX-P vs. OCD-p 0.19**<br>**SPX-P vs. EOS-P 0.65**<br>**SPX-P vs. MR-P 0.29**
**Schedule for Affective Disorders and Schizophrenia - Lifetime Version (SADS-L)**	Anxiety	USA	Piven et al (1991) [68]	ASD-P n = 81 (42Mo/39Fa)	DS-P n = 34 (18Mo/16Fa)	**ASD-P > DS-P***	
	Major Depressive Disorder	USA	Piven et al (1991) [68]	ASD-P n = 81 (42Mo/39Fa)	DS-P n = 34 (18Mo/16Fa)	**n.s**	
**Schedule for Affective Disorders and Schizophrenia -**	Major Depressive Disorder	USA	Piven & Palmer (1999) [69]	MPX-P n= 48 (25Mo/23Fa)	DS-P n = 60 (30Mo/30Fa)	**ASD-P > DS-P****	

**Lifetime Version Modified for the Study of Anxiety Disorders, Revised (SADS-LA-R)**	Social phobia	USA	Piven & Palmer (1999) [69]	MPX-P n= 48 (25Mo/23Fa)	DS-P n = 60 (30Mo/30Fa)	**ASD-P > DS-P***	
**Parental Qestionnaire (Devised by Author and results validated by consented medical records from GP)**	Depression	UK	Micali et al (2004) [70]	ASD-P n = 152 (79Mo/73Fa)	ODP-P n = 114 (59Mo/55Fa)	**ASD-Mo > ODP-Mo***	
	Anxiety	UK	Micali et al (2004) [70]	ASD-P n = 152 (79Mo/73Fa)	ODP-P n = 114 (59Mo/55Fa)	**ASD-Mo > ODP-Mo*****	
**The Centre for Epidemiological Studies – Depression Scales (CESD)**	Depressed Mood	USA	Ingersoll et al (2011) [25]	ASD-Mo n = 71 (Only Mo)	N-Mo n = 94 (Only Mo)	**ASD-Mo > N-Mo***	**ASD-Mo vs. N-Mo 0.35**
**Beck Depression Inventory (BDI)**	Depression	UK	Berthoz et al (2013) [40]	ASD-P n = 87 (28%Fa)	UA n = 47 (62%M)	**n.s.**	**ASD-P vs. UA 0.50**
**State-Trait Anxiety Inventory Form Y (STAI-Y)**							
State scale (STAI-S)	Anxiety (State portion)	UK	Berthoz et al (2013) [40]	ASD-P n = 87 (28%Fa)	UA n = 47 (62%M)	**n.s.**	**ASD-P vs. UA 0.19**
Trait scale (STAI-T)	Anxiety (Trait portion)	UK	Berthoz et al (2013) [40]	ASD-P n = 87 (28%Fa)	UA n = 47 (62%M)	**n.s.**	**ASD-P vs. UA 1.24**

Note: ASD, Autism Spectrum Disorder; BAP, Broad Autism Phenotype; BAP(+), BAP present; BAP (-), BAP absent; P, Parent; Mo, Mother; Fa, Father; M, Male; F, Female; MPX, Multiple incidence autism families; SPX, Single incidence autism families; DS, Down Syndrome; EOS, Early onset Schizophrenia; MR, Mental Retardation; N, Normative sample; OCD, Obsessive Compulsive Disorder; ODP, Other developmental problems without autism; UA, Unaffected adult

*p<0.05; **p<0.01; ***p<0.001
